# Comparative proteomic analysis of *Nicotiana benthamiana* plants under *Chinese wheat mosaic virus* infection

**DOI:** 10.1186/s12870-021-02826-9

**Published:** 2021-01-19

**Authors:** Long He, Peng Jin, Xuan Chen, Tian-Ye Zhang, Kai-Li Zhong, Peng Liu, Jian-Ping Chen, Jian Yang

**Affiliations:** 1 College of Plant Protection, Nanjing Agricultural University, Nanjing, 210095 China; 2 State Key Laboratory for Quality and Safety of Agro-products, Institute of Plant Virology, Ningbo University, Ningbo, 315211 China

**Keywords:** *Chinese wheat mosaic virus*, Differentially expressed proteins, ABA, *Nicotiana benthamiana*

## Abstract

**Background:**

*Chinese wheat mosaic virus* (CWMV) is a severe threat to winter wheat and is transmitted by *Polymyxa graminis*. The mechanisms of interactions between CWMV and plants are poorly understood. In this study, a comparative proteomics analysis based on nanoliquid chromatography mass spectrometry (MS)/MS was conducted to characterize proteomic changes in plants responding to CWMV infection.

**Results:**

In total, 2751 host proteins were identified, 1496 of which were quantified and 146 up-regulated and 244 down-regulated proteins were identified as differentially expressed proteins (DEPs). Kyoto Encyclopedia of Genes and Genomes (KEGG) enrichment analysis showed that DEPs were most strongly associated with photosynthesis antenna proteins, MAPK signaling plant and glyoxylate and dicarboxylate metabolism pathways. Subcellular localization analysis predicted that more than half of the DEPs were localized in the chloroplast, an organelle indispensable for abscisic acid (ABA) synthesis. Our results suggest that CWMV infection interrupts normal chloroplast functions and decreases ABA concentrations in *Nicotiana benthamiana*. Further analysis showed that the ABA pathway was suppressed during CWMV infection and that ABA treatment induced plant hosts defenses against CWMV.

**Conclusions:**

We identified several candidate proteins expressed during CWMV infection, and the ABA pathway was strongly associated with responses to CWMV infection in *N. benthamiana*.

**Supplementary Information:**

The online version contains supplementary material available at 10.1186/s12870-021-02826-9.

## Background

Viral infections have severely reduced yield and quality of different crop plants in the past decades [1, 2]. Plant host resources and factors are indispensable for viral infection as plant viruses have small genomes that encode relatively few proteins [3, 4]. Increasing evidence suggests that a variety of host factors are required in various steps of virus infection. For instance, *rice dwarf virus* (RDV) infection subverts auxin signaling in rice as the interaction between the proteins RDV P2 and OsIAA10 disrupts interactions between OsIAA10 and OsTIR1 [5]. P5–1 protein encoded by *rice black streak dwarf virus* (RBSDV) physically interacts with OsCSN5A, which interferes with the ubiquitination activity of SCF E3 ligase and suppresses jasmonate signaling to facilitate viral infection of rice [6].

Wheat (*Triticum aestivum* L.) is one of the most important food crops for humans worldwide [7, 8]. However, wheat quality and yield are limited by many unfavorable factors including biotic and abiotic stressors [9]. A number of soil-borne viruses can infect wheat under natural conditions and thereby affect wheat growth and development [10–12]. *Chinese wheat mosaic virus* (CWMV) is a soil-borne virus that was first described in China [13]. CWMV is identified as an member of the genus *Furovirus* in the family *Virgaviridae*, and its genome comprises the two positive sense single-stranded RNAs, RNA1 and RNA2 [14–17]. CWMV RNA1 consists of 7147 nucleotides (nt) and has three major predicted open reading frames that encode three proteins necessary for viral replication and transmission. CWMV RNA2 is 3564 nt long and encodes four proteins: the major coat protein (CP; 19 kDa), two CP-related proteins (N-CP; 23 kDa and CP-RT, 84 kDa) and a cysteine-rich protein (CRP; 19 kDa), that functions as an RNA silencing suppressor [18–20]. So far, only a few studies have focused on the relationships between CWMV and its host. Silencing of *NbRDR6* reduces CWMV accumulation and siRNAs at higher temperatures [21]. NbHSP70 interacts with CWMV RNA1-encoded viral replicase, and its subcellular localization was changed due to CWMV replicase action [22]. CWMV-derived vsiRNA-20 interferes with the content of H^+^ in viral infected cells, resulting in CWMV accumulation [23]. Identifying potential host factors participating in plant interactions with CWMV during infection is an urgent matter.

Quantitative proteomics have been developed and commonly applied in plant-microbe interaction studies. For example, through quantitative whole-proteome analysis, Fu et al. found that the expression of a remorin protein (NbREM1) is suppressed under *rice stripe virus* (RSV) infection, and further research revealed that RSV-encoded NSvc4 protein interacts with NbREM1, and it inhibits NbREM1 S-acylation to facilitate RSV infection [24]. Proteomic analysis was performed to address the effects of RBSDV on maize protein abundance, which revealed that RBSDV infection in maize is regulated by a number of metabolic pathways [25]. Maize cv. B73 plants infected with *maize chlorotic mottle virus* (MCMV) has been investigated in a comparative proteomic study to produce detailed whole-proteome information showing that the expression levels of ribosomal proteins, proteins related to stress responses, oxidation-reduction processes, and redox homeostasis are significantly altered during MCMV infection [26]. *Nicotiana benthamiana* is the most commonly experimental model in molecular plant-microbe interaction studies because it is highly susceptible to multiple pathogens and amenable to transient protein expression and virus-induced gene silencing [27]. Heat shock protein 70 (HSP70) has been reported to be required for RSV infection in both rice, which is the natural host, and *N. benthamiana,* an experimental host [28]. The protein γb of *barely stripe mosaic virus* is a multifunctional protein that can inhibit autophagy by interfering with interactions between AUTOPHAGY PROTEIN (ATG)7 and ATG8 in *N. benthamiana* as an experimental host [29]. Moreover, artificial full-length CWMV cDNA clones that can successfully infect *N. benthamiana* have been developed, which is of considerable interest for subsequent research [30, 31]. Some progress has been made regarding our understanding of the elaborate interactions between CWMV and its plant host; however, no studies on the responses of plant hosts to CWMV infection at the proteomic level were available so far.

In the present study, we performed comparative proteomic analysis of *N. benthamiana* plants during CWMV infection. A certain number of differentially expressed proteins (DEPs) were identified. We also found CWMV infection suppressed the abscisic acid (ABA) signaling pathway, whereas ABA application induced *N. benthamiana* resistance against CWMV. Combined with virus-induced gene silencing (VIGS) technology, we found that silencing of the genes encoding zeaxanthin epoxidase (NbABA1) or xanthoxin dehydrogenase (NbABA2) might increase CWMV accumulation, probably by interfering the ABA pathway. Our results suggest candidate factors for cultivation of resistant wheat varieties and provide new insights into the molecular basis of CWMV pathogenicity.

## Results

### Overview of quantitative proteomic analyses

**Fig. 1:**
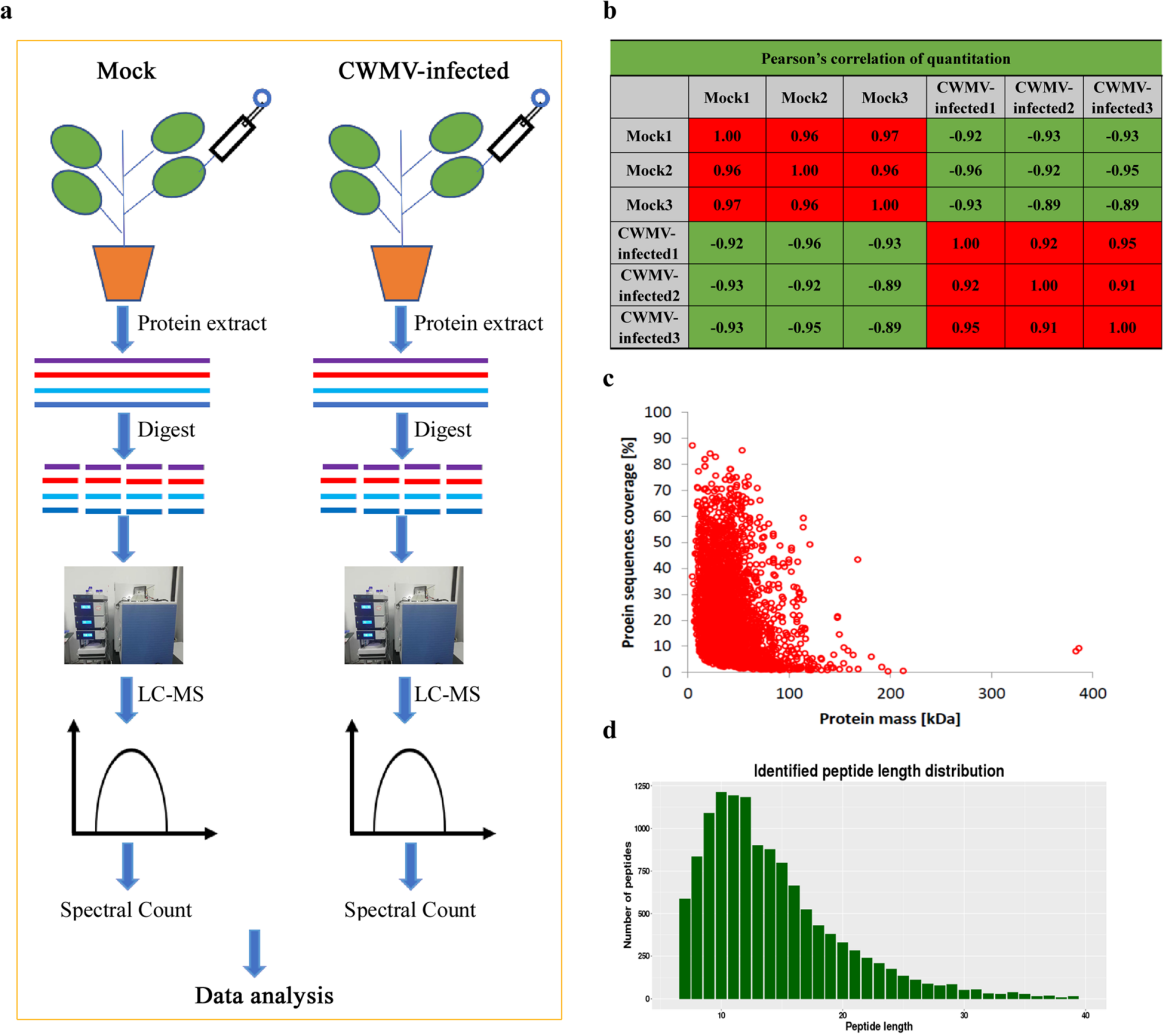
Experimental strategy for quantitative proteome analysis and quality control validation of MS data. **a** Proteins were extracted from three biological replicates of each sample group. All protein samples were digested with trypsin and analyzed by LC/MS. **b** Pearson’s correlations. **c** Mass delta of all identified peptides. **d** Length distribution of all identified peptides

Fourteen days post inoculation of four-leaf stage plants with CWMV, several chlorotic lesions appeared on the upper leaves of the inoculated plants (Additional file 1: Fig. S1a). In addition, CWMV infection was confirmed by reverse transcription-polymerase chain reaction (RT-PCR) and western blotting (Additional file 1: Fig. S1b and c). Symptomatic leaves were collected for quantitative proteomic analysis. This analysis was performed to investigate proteomic changes in CWMV-infected plants compared to control plants; the workflow is shown in Fig. 1a. Pearson’s correlation coefficients indicated that biological repeat correlations were sufficient (Fig. 1b). In total, 12,845 peptides were detected, and the average mass error was < 0.02 Da, indicating high mass accuracy of mass spectrometry (MS) data (Fig. 1c). The lengths of most identified peptides were distributed from 7 to 20 amino acid residues, which was consistent with the rules based on trypsin enzymatic hydrolysis and HCD fragmentation (Fig. 1d), and thus, met the quality control standard. Detailed information on identified peptides is provided in Additional file 2: Table S1. In total, 2751 proteins were identified, 1496 of which were quantified. To further study the function of identified proteins, these proteins were annotated respectively according to GO terms, subcellular localizations, Kyoto Encyclopedia of Genes and Genomes (KEGG) pathways, and predicted functional domains. Detailed information of identified proteins is listed in Additional file 3: Table S2.

### Impact of CWMV infection on the *N. benthamiana* proteome

**Fig. 2:**
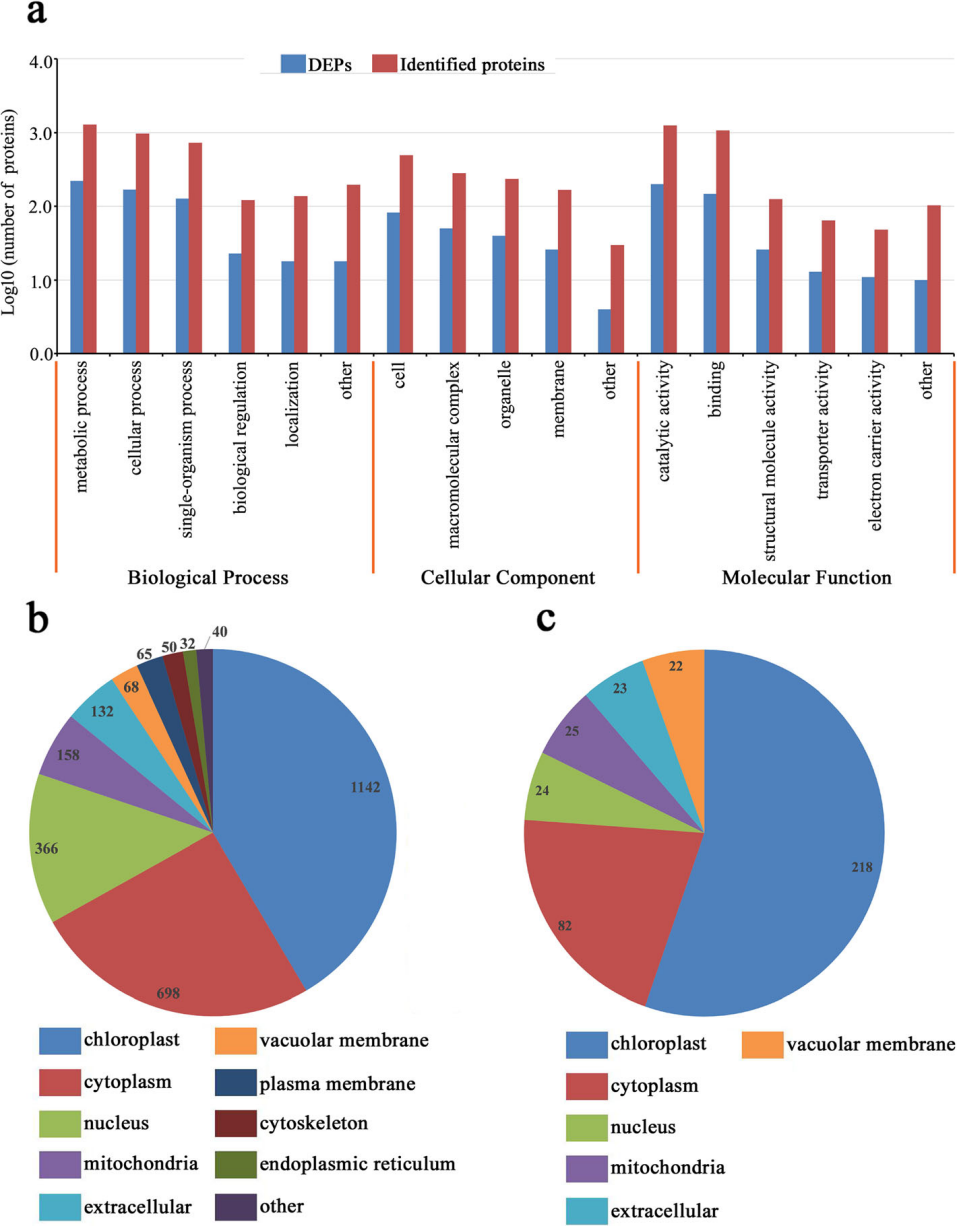
Classifications of identified proteins and DEPs. **a** GO analysis of all identified proteins and DEPs. **b** Subcellular locations analysis of all identified proteins. **c** Subcellular locations analysis of all DEPs

Proteins with a mean fold-change>1.5 or<0.67 at *P*<0.05 were considered differentially expressed proteins (DEPs). Out of the 1496 quantified proteins, 390 proteins were identified as DEPs. Detailed information on DEPs is listed in Additional file 4: Table S3. According to the results of Gene Ontology (GO) analysis, 2751 identified proteins and 390 DEPs were categorized into three major groups: biological processes, cellular components, and molecular functions (Fig. 2a). In the biological processes group, 1290 identified proteins and 221 DEPs were involved in metabolic processes, and 972 identified proteins and 168 DEPs were involved in cellular processes; in the cellular components group, 493 identified proteins and 82 DEPs were associated with the cell, and 281 identified proteins and 50 DEPs were associated with macromolecular complexes; and in the molecular functions group, 1253 identified proteins and 200 DEPs were involved in catalytic activities, and 1072 identified proteins and 147 DEPs were involved in binding activities.

All identified proteins and DEPs were categorized according to their subcellular localizations. In summary, 15 different subcellular components were identified, including 1142 chloroplast-localized proteins, 698 cytoplasm-localized proteins, and 366 nucleus-localized proteins (Fig. 2b). Regarding DEPs, only 10 different subcellular components were found, including 218 chloroplast-localized DEPs comprising 52 up-regulated and 166 down-regulated DEPs, 82 cytoplasm-localized DEPs comprising 36 up-regulated and 46 down-regulated DEPs, and 24 nucleus-localized DEPs comprising 13 up-regulated and 11 down-regulated DEPs (Fig. 2c).

### Enrichment analysis of DEPs in response to CWMV infection

**Fig. 3:**
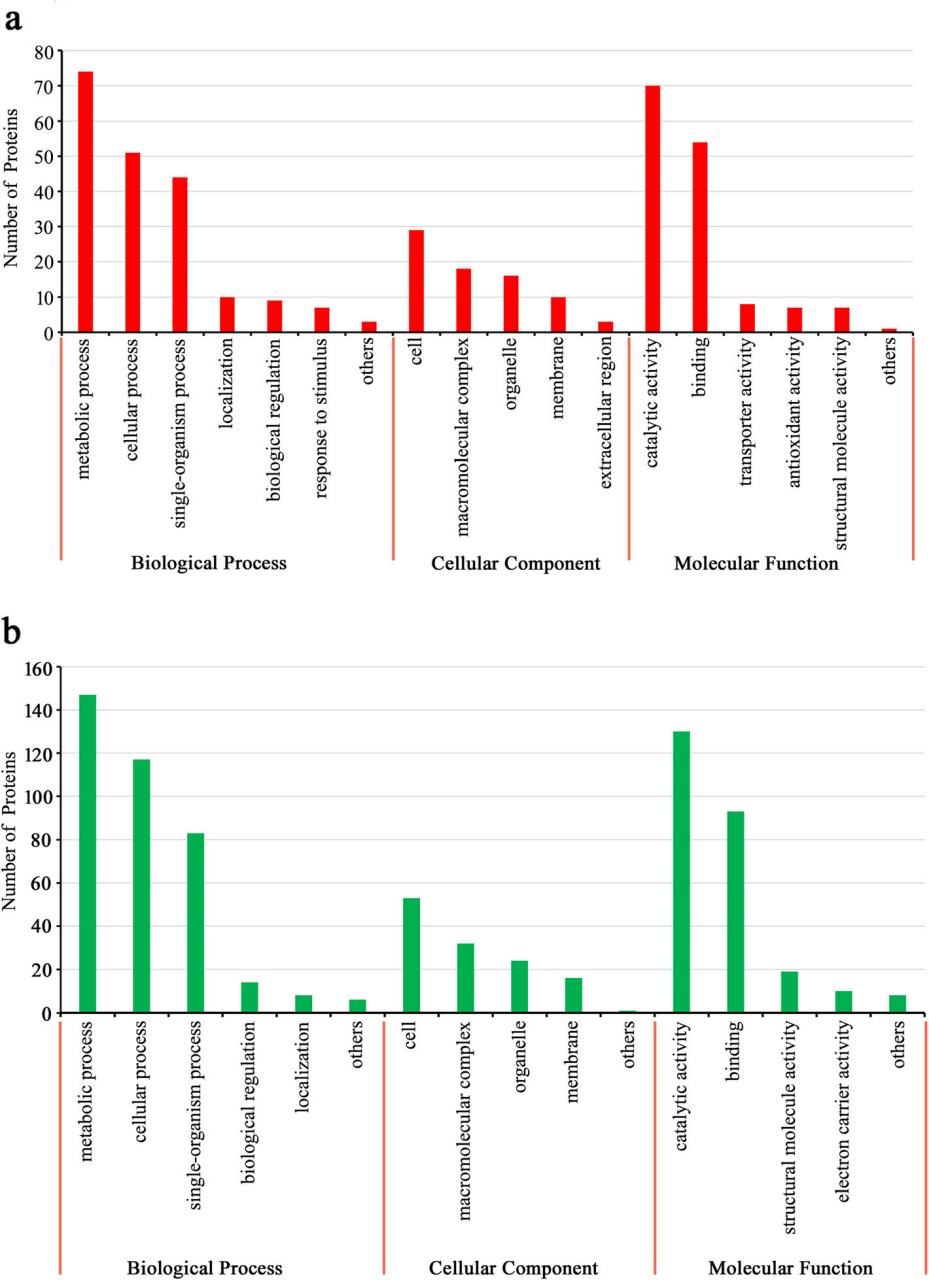
GO analysis of all DEPs. **a** GO analysis of up-regulated DEPs. **b** GO analysis of down-regulated DEPs

**Fig. 4:**
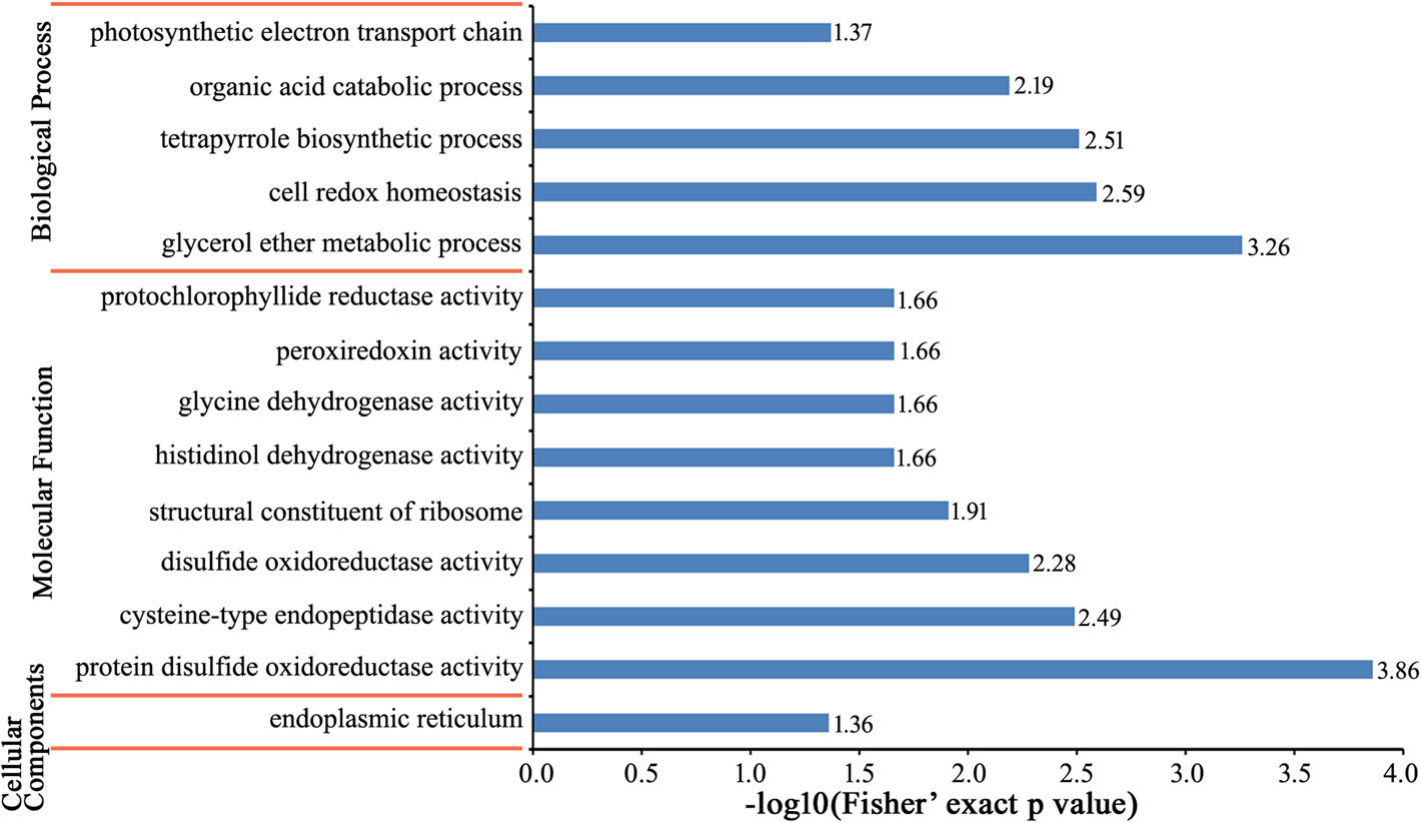
GO enrichment analysis of DEPs. Significantly enriched GO terms of all DEPs

**Fig. 5:**
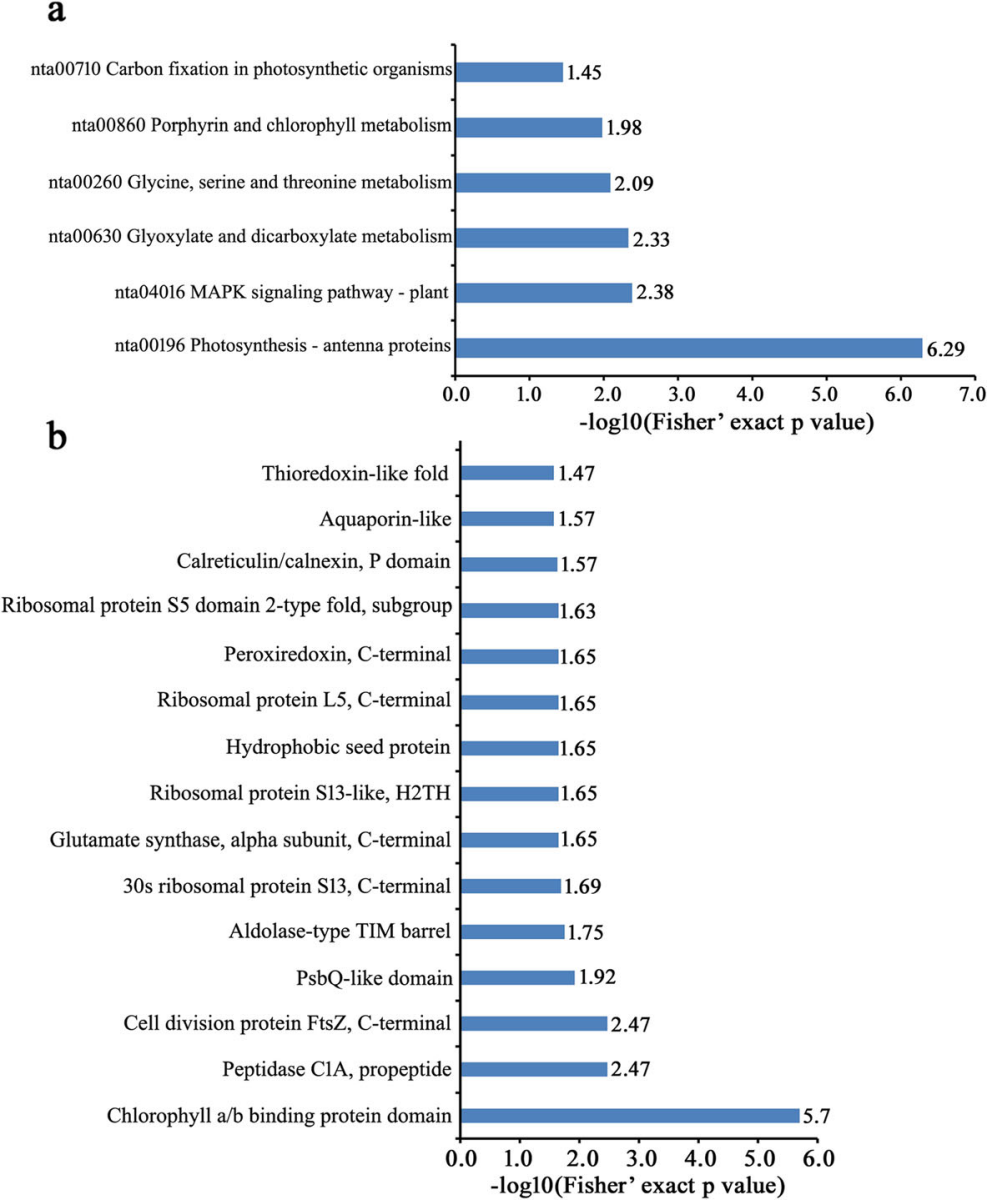
Enrichment analysis of DEPs. **a** Significantly enriched KEGG terms of all DEPs. **b** Significantly enriched protein domain terms of all DEPs

In order to further explore DEPs as a response to CWMV infection, enrichment analysis of 390 DEPs was performed based on GO annotations, KEGG analysis, and protein domains. Among these 390 DEPs, 146 proteins were significantly up-regulated, and 244 proteins were significantly down-regulated (Additional file 5: Fig. S2). A large number of up-regulated DEPs were mainly associated with metabolic processes (74 proteins), catalytic activities (70 proteins), and binding (54 proteins) (Fig. 3a). Regarding down-regulated DEPs, metabolic processes (147 proteins), catalytic activities (130 proteins), and cellular processes (117 proteins) were predominant (Fig. 3b). In the GO enrichment-based cluster analysis, DEPs involved in three functional groups were plotted. DEPs in the biological processes group were enriched in glycerol ether metabolic processes, cell redox homeostasis, tetrapyrrole biosynthetic processes, organic acid catabolic processes, and in the photosynthetic electron transport chain. DEPs in the molecular function group were enriched in protein disulfide oxidoreductase activity, cysteine-type endopeptidase activity, disulfide oxidoreductase activity, as structural constituents of the ribosome, histidinol dehydrogenase activity, glycine dehydrogenase (decarboxylating) activity, peroxiredoxin activity, and protochlorophyllide reductase activity. DEPs in the cellular components group were enriched in the endoplasmic reticulum (Fig. 4). KEGG enrichment analysis showed that DEPs were closely related to photosynthesis antenna proteins (nta00196), the plant MAPK signaling pathway (nta04016), glyoxylate and dicarboxylate metabolism (nta00630), glycine, serine and threonine metabolism (nta00260), porphyrin and chlorophyll metabolism (nta00860), and carbon fixation in photosynthetic organisms (nta00710) (Fig. 5a). Protein enrichment analysis showed that DEPs were mainly enriched in the chlorophyll a/b-binding protein domain, peptidase C1A, propeptide, cell division protein FtsZ, C-terminal, PsbQ-like domain, aldolase-type TIM barrel, thioredoxin domain, 30S ribosomal protein S13, C-terminal, glutamate synthase, alpha subunit, C-terminal, ribosomal protein S13-like protein, H2TH, hydrophobic seed protein, ribosomal protein L5, C-terminal, peroxiredoxin, C-terminal, ribosomal protein S5 domain 2-type fold, subgroup, calreticulin/calnexin, P domain, aquaporin-like, thioredoxin-like fold, glyceraldehyde 3-phosphate dehydrogenase, NAD(P) binding domain, glyceraldehyde 3-phosphate dehydrogenase, catalytic domain, alpha-D-phosphohexomutase alpha/beta/alpha domain III, and alpha-D-phosphohexomutase alpha/beta/alpha domain I (Fig. 5b).

### Identification of representative DEPs from the three most enriched KEGG pathways

According to our proteomic data analysis, a total of 15 DEPs was involved in the photosynthesis antenna proteins pathway, 9 DEPs were involved in the plant MAPK signaling pathway, and 18 DEPs were involved in the glyoxylate and dicarboxylate metabolism pathway. Five representative proteins from each the photosynthesis antenna proteins pathway (P27491, Q0PWS6, A0A1S3YAU9, Q0PWS5, and A0A1S4CBW5), from the plant MAPK signaling pathway (P17514, A0A1S3XTH4, P24091, A0A1S3ZVW5, and A0A1S4DGP1), and from the glyoxylate and dicarboxylate metabolism pathway (A0A1S3YRT4, A0A1S3ZFE6, A0A1S3Y2X0, A0A1S3YYG2, and A0A1S4BAT9) were examined regarding the three pathways that were most significantly enriched (Table 1).

**Table 1:** Identification of selected DEPs from significantly changed pathways through KEGG enrichment analysis

Protein accession	Protein description	Infected/Mock Ratio	Infected/Mock ***P*** value	Subcellular localization
Photosynthesis - antenna proteins				
P27491	Chlorophyll a-b binding protein 7	0.132	0.0000002020	chloroplast
Q0PWS6	Chlorophyll a-b binding protein	0.279	0.000078452	chloroplast
A0A1S3YAU9	Chlorophyll a-b binding protein	0.359	0.00000057805	chloroplast
Q0PWS5	Chlorophyll a-b binding protein	0.361	0.0000027605	chloroplast
A0A1S4CBW5	Chlorophyll a-b binding protein	0.397	0.000023582	chloroplast
MAPK signaling pathway – plant				
P17514	Acidic endochitinase Q	48.587	0.008995	nucleus
A0A1S3XTH4	Basic form of pathogenesis-related protein 1-like	2.983	0.000075712	chloroplast
P24091	Endochitinase B	2.863	0.000121412	extracellular
A0A1S3ZVW5	Basic endochitinase-like	2.264	0.0138624	chloroplast
A0A1S4DGP1	Nucleoside diphosphate kinase	0.472	0.0094373	chloroplast
Glyoxylate and dicarboxylate metabolism				
A0A1S3YRT4	Glycine cleavage system H protein	4.204	0.0021605	mitochondria
A0A1S3ZFE6	Catalase	1.53	0.0082996	cytoplasm
A0A1S3Y2X0	Serine hydroxymethyltransferase	0.43	0.0047004	mitochondria
A0A1S3YYG2	Glycine cleavage system P protein	0.444	0.00091813	mitochondria
A0A1S4BAT9	Glutamate--glyoxylate aminotransferase 2	0.531	0.000020175	cytoplasm

### Transcriptional level analysis of selected DEPs

**Fig. 6:**
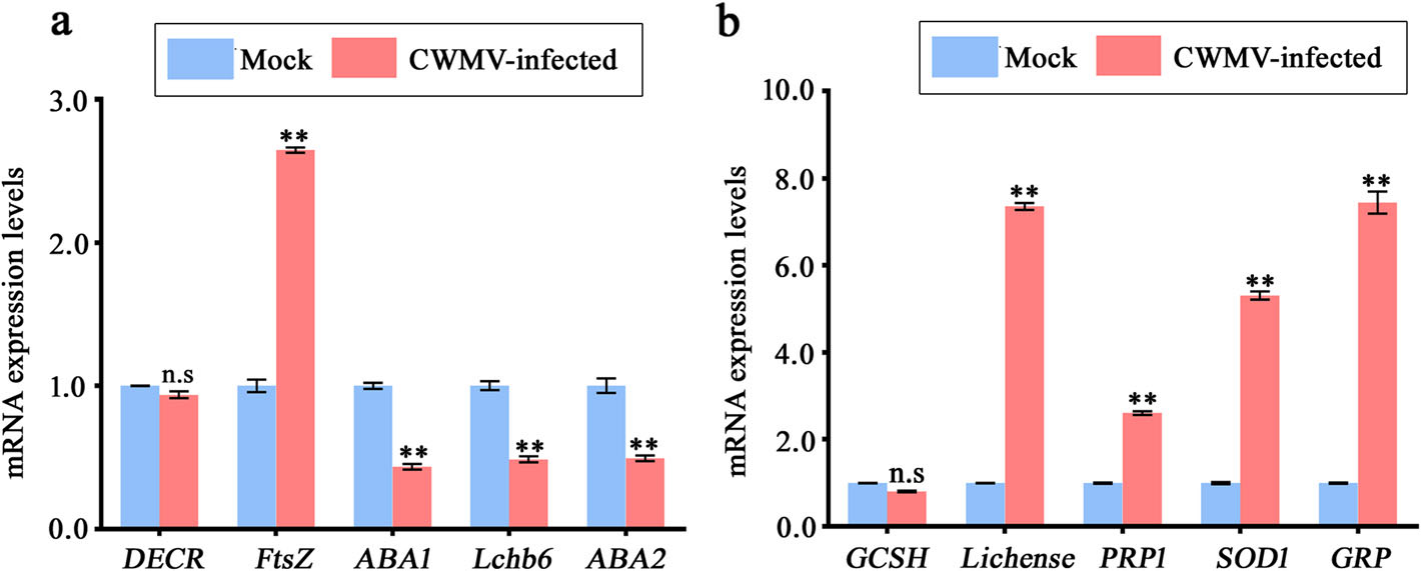
Transcriptional analyses of selected DEPs. **a** RT-qPCR analyses of genes encoding the selected down-regulated DEPs in controls and CWMV-infected plants. **b** RT-qPCR analyses of genes encoding the selected up-regulated DEPs in controls and CWMV-infected plants. Control plants were agro-infiltrated with agrobacterium cultures carrying the empty vector pCB-35S. Means ± standard deviations (SE) were calculated from three biological replicates relative to control plants, and each biological replicate comprised three technical replicates. ns, not significant; **, *P* < 0.01 (Student’s *t*-test). Controls were agro-infiltrated with agrobacterium cultures carrying the empty vector pCB-35S

To verify changes at the protein level as determined by proteomic analysis, five genes encoding up-regulated DEPs and five genes encoding down-regulated DEPs were randomly selected for quantitative reverse transcription polymerase chain reaction (RT-qPCR). Expression levels of three of the selected genes encoding down-regulated DEPs (Genebank accession numbers XM_016620556, XM_016614219, and XM_016584441) were decreased (Fig. 6a). No significant change in the expression levels of the *NbDECR* gene (Genebank accession number XM_016603860) was observed, and expression levels of the *NbftsZ* gene (Genebank accession number XM_016656262) were significantly increased. Among the selected genes encoding up-regulated DEPs, no significant change of *NbGCSH* (Genebank accession number XM_016599276) expression was observed while expression levels of the other genes (Genebank accession numbers XM_016640264, XM_016599276, XM_016631233, and XM_016645028) were increased during CWMV infection (Fig. 6b). Numerous studies have reported that gene transcription and protein abundance are not correlated; however, our results suggest that quantities of mRNA of genes encoding most DEPs are consistent with their protein expression levels. All basic information on selected DEPs is listed in Additional file 6: Table S4.

### ABA pathway suppression in CWMV-infected plants

Subcellular localization analysis showed that 35.62% of the up-regulated DEPs and 66.94% of the down-regulated DEPs were predicted to be localized in the chloroplast (Additional file 7: Fig. S3). These results suggested that CWMV invasion probably altered the chloroplasts’ functions. Synthesis of various plant hormones including ABA is closely regulated by the chloroplast machinery system [32]. Furthermore, label-free profiling analysis revealed that expression levels of ABA1 and ABA2, which are involved in the ABA-pathway, were significantly down-regulated in CWMV-infected plants, compared to control plants. Furthermore, mRNA expression of *NbABA1* and *NbABA2* was also significantly suppressed in CWMV-infected plants, compared to control plants (Fig. 6a).

**Fig. 7:**
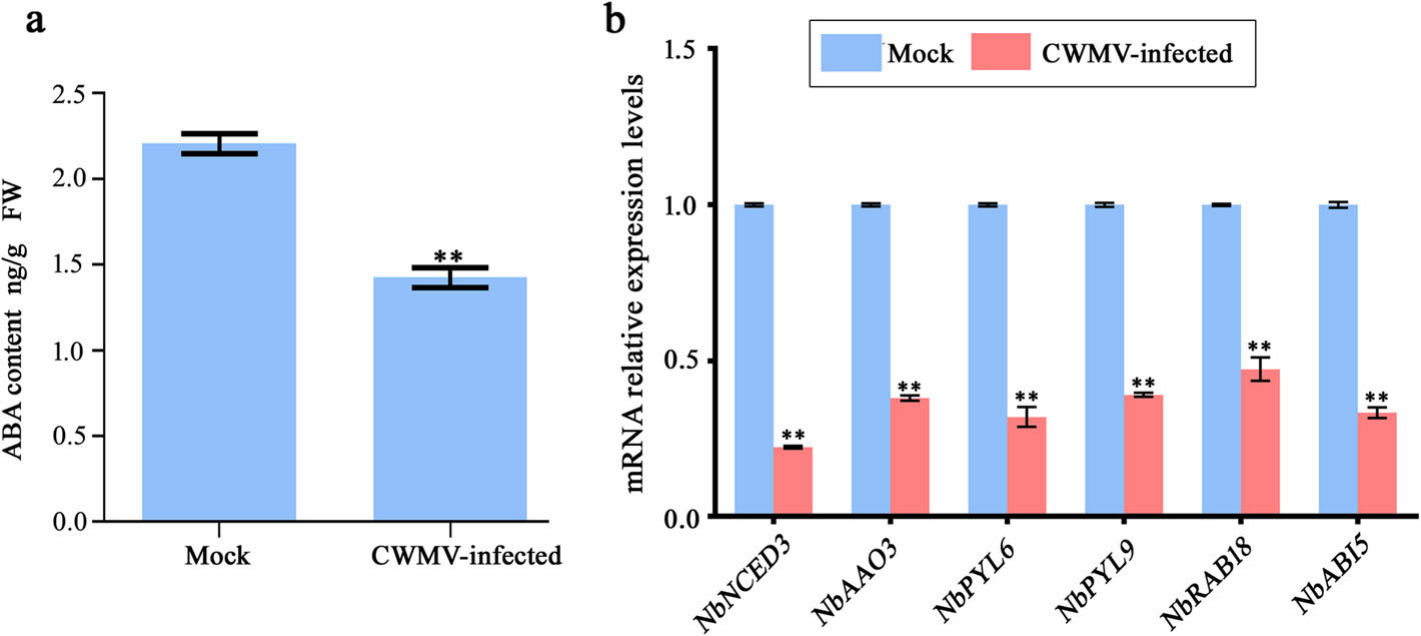
Effects of CWMV infection on the ABA pathway. **a** ABA concentrations in controls and CWMV-infected plants. Three independent biological replicates were analyzed for each treatment. **, *P* < 0.01 determined by Student’s *t*-test. **b** RT-qPCR analysis of ABA*-*pathway genes in control and CWMV-infected plants. Means ± SE were calculated from three biological replicates relative to control plants, and each replicate had three technical replicates. **, *P* < 0.01 (Student’s *t*-test). Control plants were agro-infiltrated with agrobacterium cultures carrying the empty vector pCB-35S

To investigate the effects of CWMV infection, we measured ABA concentrations in *N. benthamiana* plants, which showed that the ABA content was significantly decreased in CWMV-infected plants, compared with that in control plants (Fig. 7a). In addition, RT-qPCR analysis indicated that mRNA expression levels of ABA-biosynthetic genes (*NbNCED3* and *NbAAO3*; Genebank accession numbers GQ477382 and EH364870, respectively), ABA signaling transduction genes including (*NbPYL6* and *NbPYL9*; Genebank accession numbers TC19819 and EH365959, respectively), ABA-responsive genes (*NbRAB18* and *NbABI5*; Genebank accession numbers Q6DHC1 and Q9SJN0, respectively) were significantly down-regulated in CWMV-infected plants, compared with expression levels in control plants (Fig. 7b). Taken together, these results implied that the ABA pathway was substantially altered in response to CWMV infection.

### Knockdown of ABA1 AND ABA2 enhanced CWMV accumulation

**Fig. 8:**
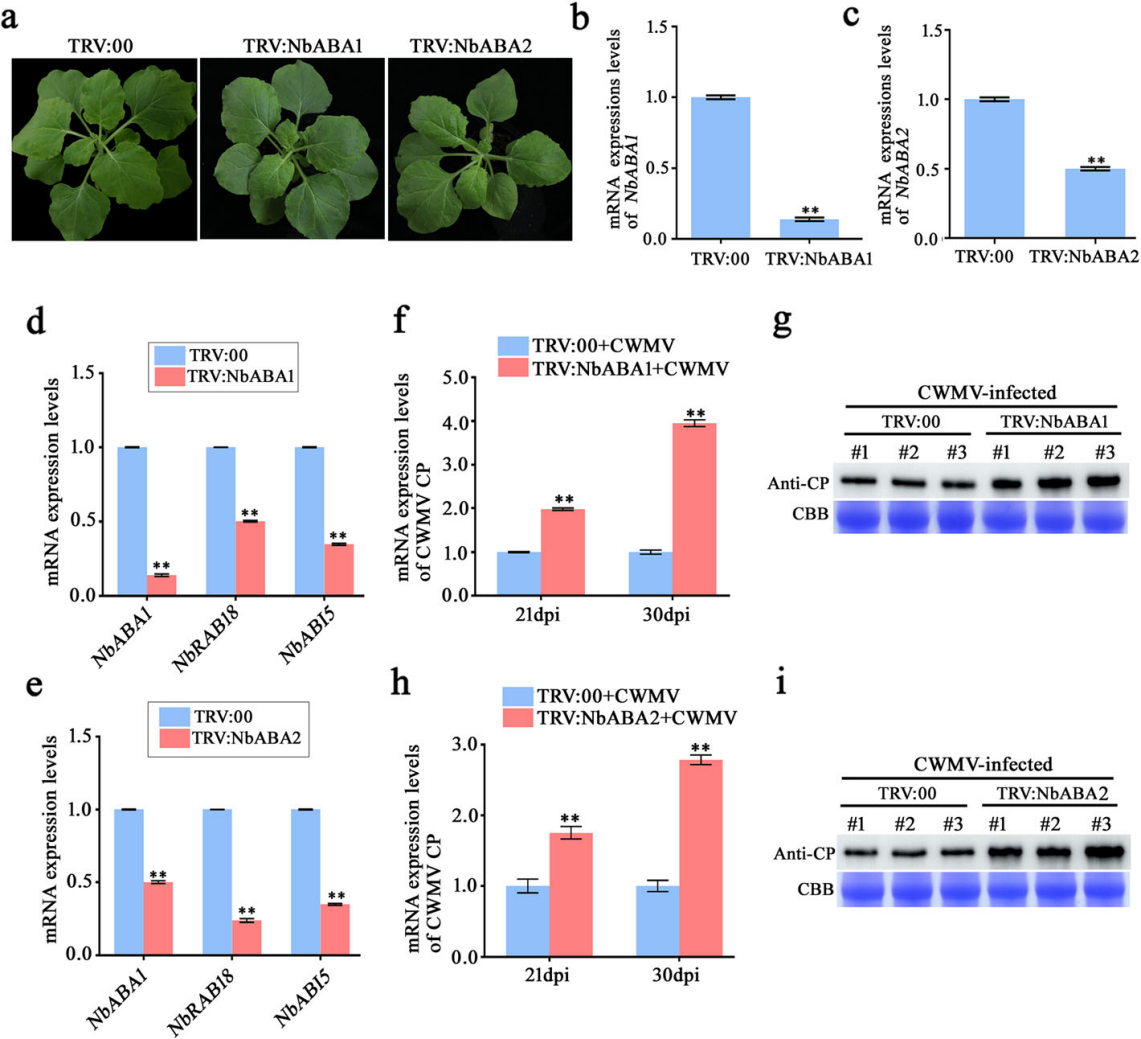
Effects of silencing *NbABA1* or *NbABA2* on CWMV CP accumulation. **a** Phenotypes of *NbABA1*-silenced and *NbABA2*-silenced plants. Photograph taken 7 d post agro-infiltration. **b** RT-qPCR results of relative expression levels of *NbABA1* and *NbABA2*. **c** RT-qPCR results of relative expression levels of *NbRAB18* and *NbABI5* in *NbABA1*-silenced plants. **d** RT-qPCR results of relative expression levels of *NbRAB18* and *NbABI5* in *NbABA2*-silenced plants. Means ± SE were calculated from three biological replicates relative to TRV:00 plants, and each replicate comprised three technical replicates. **, *P* < 0.01 (Student’s *t*-test). **e** RT-qPCR results of mRNA expression of CWMV CP in *NbABA1*-slienced plants. Means ± SE were calculated from three biological replicates relative to CWMV-infected TRV:00 plants, and each replicate comprised three technical replicates. **, *P* < 0.01 (Student’s *t*-test). **f** Western blot showing protein expression of CWMV CP in *NbABA1*-slienced plants. Coomassie brilliant blue-stained loadings are shown in the lower section of the figure. **g** RT-qPCR showing mRNA expression of CWMV CP in *NbABA2*-silenced plants. Means ± SE were calculated from three biological replicates relative to CWMV-infected TRV:00 plants, and each replicate comprised three technical replicates. **, *P* < 0.01 (Student’s *t*-test). **h** Western blot assay showing protein expression of CWMV CP in *NbABA2*-slienced plants. Coomassie brilliant blue-stained loadings are shown in the lower section of the figure

The relationship between the ABA pathway and viral infection has been studied to some extent; however, the roles of ABA pathway-related genes regarding ABA-CWMV interactions remain unclear. Therefore, to investigate the functions of NbABA1 and NbABA2 during CWMV infection, we silenced *NbABA1* and *NbABA2* and inoculated these modified plants with CWMV. Amino acid sequence analyses showed that *NbABA1* shared 96% sequence identity with *N. tabacum* ABA1 (NtABA1) and 69% sequence identity with *Arabidopsis thaliana* ABA1 (AtABA1) (Additional file 8: Fig. S4). *NbABA2* shared a 92% sequence identity with *N. tabacum* ABA2 (NtABA2) and 66% sequence identity with *A. thaliana* ABA2 (AtABA2) (Additional file 9: Fig. S5). Thus, an approximately 250-nt sequence in the conserved domain of *NbABA1* was chosen for silencing *NbABA1*. The same method was used for *NbABA2* silencing. RT-qPCR data confirmed that *NbABA1* and *NbABA2* mRNA expression levels were down-regulated (Fig. 8b and c). No significant phenotypic changes were observed in *NbABA1*-silenced or *NbABA2*-silenced plants (Fig. 8a). Moreover, the RT-qPCR results showed that expression levels of ABA-responsive genes including *NbRAB18* and *NbABI5* were down-regulated in *NbABA1*-silenced and *NbABA2* -silenced plants, compared to those in *tobacco rattle virus* (TRV):00-treated plants (Fig. 8d-e). These results, in combination with amino acid sequence analysis, confirm that NbABA1 and NbABA2 are indeed involved in the ABA signaling pathway.

We then inoculated *NbABA1*-silenced and *NbABA2*-silenced plants with CWMV. TRV:00-treated plants inoculated with CWMV were used as controls. Twenty-one days post inoculation (dpi) with CWMV, RT-qPCR and western blotting showed that accumulation levels of CWMV CP RNA and of CP were higher in *NbABA1*-silenced (Fig. 8f and g) and *NbABA2*-silenced (Fig. 8h and i) plants, compared to those in the controls. In addition, 30 days post inoculation (dpi) with CWMV, RT-qPCR analyses showed that accumulation levels of CWMV CP RNA were much higher in *NbABA1*-silenced (Fig. 8f) and *NbABA2*-silenced (Fig. 8h) plants, compared to those in the controls. Taken together, our results suggest that knockdown of *NbABA1* or *NbABA2* reduced plant resistance against viral infection.

### Exogenous application of ABA induces plant resistance against CWMV infection

**Fig. 9:**
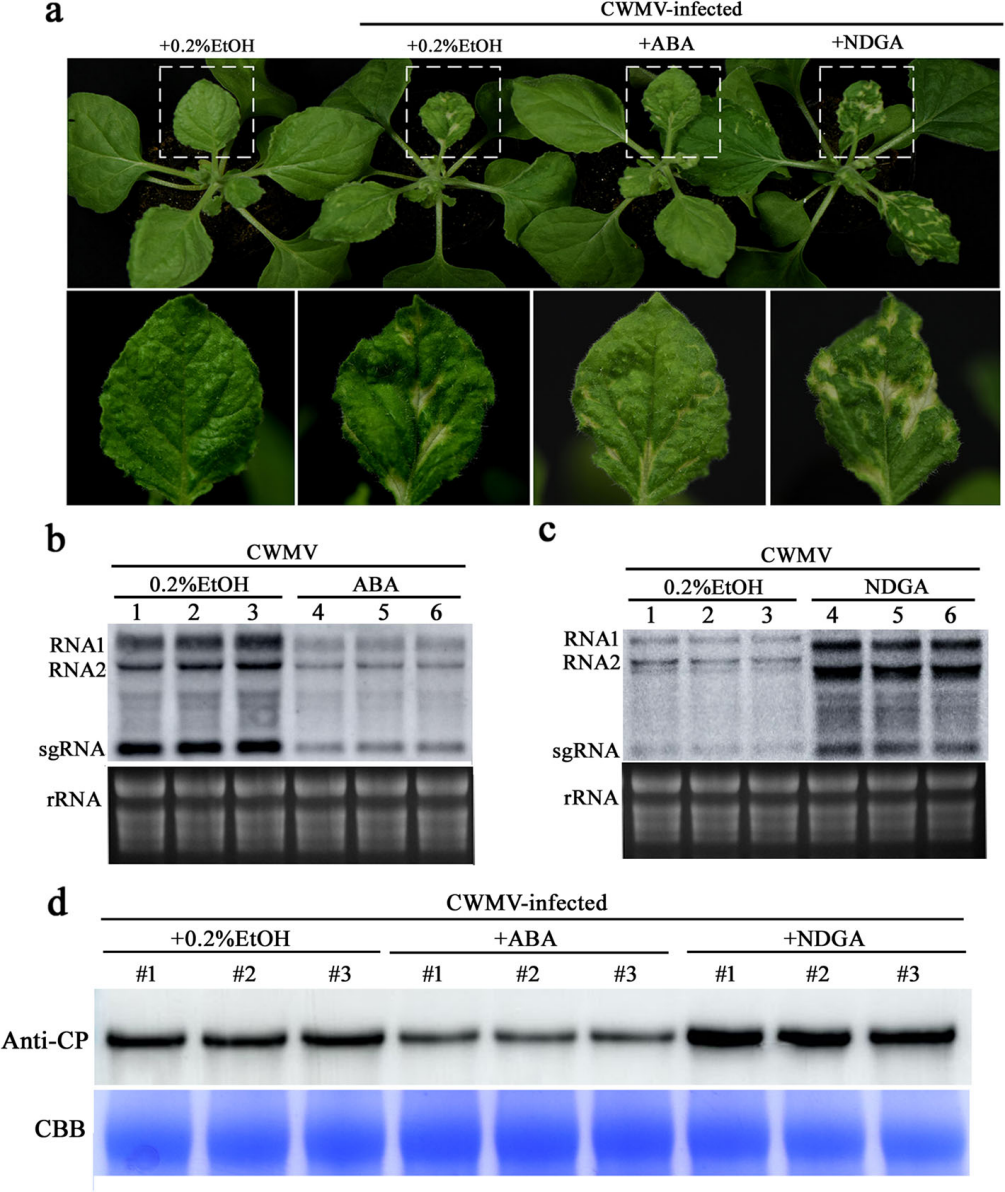
Effects of applying ABA and its inhibitor on CWMV infection in plants. **a** Phenotypes of *N. benthamiana* treated with 100 μM ABA,10 mM NDGA, or 0.2% ethanol followed CWMV inoculation at 28 d post CWMV inoculation (dpi). **b** Northern blot analyses of CWMV genomic RNAs accumulations. Samples were collected from the upper leaves of ABA-pretreated CWMV-inoculated plants. Ethidium bromide-stained rRNA was used as a loading control. **c** Northern blot analyses of genomic RNAs accumulations. Samples were collected from the upper leaves of NDGA-pretreated CWMV-inoculated plants. Ethidium bromide-stained rRNA was used as a loading control. **d** Western blot analyses of CWMV CP accumulation in ABA or NDGA pre-treated CWMV-inoculated plants. Samples were collected from the upper systemic leaves of the ABA- or NDGA- pretreated CWMV-inoculated plants. Coomassie brilliant blue-stained loadings are shown in the lower section of the figure. Controls were pre-treated with 0.2% ethanol and 12 h later were inoculated with CWMV

ABA plays a significant role in plant defense responses; we therefore treated four-leaf *Nicotiana benthamiana* with ABA (100 μM), with an ABA inhibitor (NDGA; 10 mM), or with 0.2% ethanol (control). Twelve hours post-treatment, the pre-treated plants were inoculated with CWMV. The inoculated plants were grown in a greenhouse for approximately four weeks. The results showed that control plants produced typical symptoms of CWMV infection including stunting and local chlorotic lesions, whereas ABA-treated tobacco showed only mild CWMV symptoms (Fig. 9a). As expected, NDGA-treated plants showed more substantial CWMV symptoms, and the accumulation levels of CWMV CP RNA and CP were the highest in NDGA pre-treated plants and the lowest in ABA pre-treated plants (Fig. 9b-d). Furthermore, we investigated the effects of ABA on CWMV infection in wheat, the natural CWMV host. Wheat seedings were applied with 50 μM ABA solution or 0.2% ethanol solution (control). Twelve hours post-treatment, pre-treated wheat seeding leaves were mechanically inoculated with CWMV. The outcome of RT-qPCR showed that the accumulation levels of CWMV CP RNA in ABA-treated wheat were lower than that in 0.2% ethanol-treated wheat (Additional file 12: Fig. S6). Additionally, considering effects of ABA on amount of viral RNA expressed in inoculated leaves, we conducted the following research; four- leaf stage *N. benthamiana* plants were treated with ABA and plants treated with 0.2% ethanol were regarded as controls. 12 h later, we inoculated these pre-treated plants with the agrobacterium cultures only carrying CWMV RNA2. Three days post inoculation, we collected the inoculated leaves for RT-qPCR. RT-qPCR analyses showed that there is no significant change in the expression levels of *CP* and *CRP*, which confirmed that ABA reduction is beneficial to CWMV infection (Additional file 13: Fig. S7). Taken together, our results suggest that ABA treatment can induce *N. benthamiana* defense against CWMV.

## Discussion

CWMV is transmitted by *P. graminis*, the resting spores of which can survive in soils for over 10 years, which poses a persistent threat to winter wheat production. Furthermore, *P. graminis* is also a carrying vector transmitting *wheat yellow mosaic virus*, which together with CWMV, can co-infect wheat, resulting in higher loss of quantity and quality. Thus, furthering our understanding of the molecular mechanisms underlying CWMV infection and host responses is an urgent matter. In the current study, a label-free-based comparative proteomic method was used to obtain comprehensive whole-proteome insight into CWMV infection of *N. benthamiana* plants. The outcome of our work provides new insights regarding the molecular basis of plant defense responses to CWMV.

Pearson’s correlation coefficients were strong (Fig. 1b). The relationship of protein mass and coverage, and the distribution of identified peptides are both as expected (Fig. 1c-d). These results demonstrate that the data analytical reproducibility and quality are sufficient. A total of 390 DEPs was identified from *N. benthamiana* inoculated with CWMV. Among these DEPs, 146 were up-regulated and 244 were down-regulated (Additional file 5: Fig. S2). It has been reported that the glyoxylate and dicarboxylate metabolism pathway mainly enhances plants resistance against environmental stress by balancing metabolic disorders and transferring energy [33, 34]. In the present study, we also found that glyoxylate and dicarboxylate metabolism pathway was significantly enriched after CWMV infection. Among these DEPs, 18 proteins were characterized, and their expressions levels were significantly changed, suggesting CWMV infection may destroy the host immune system by interfering with the glyoxylate and dicarboxylate metabolism pathway. The MAPK signaling pathway is strongly associated with plant growth and development, plant responses to environmental stressors, and pathogen invasion [35]. Payne et al. cloned two acidic endochitinase genes encoding the pathogenesis-related proteins PR-P and PR-Q, and expression of these two proteins was found to be induced following *tobacco mosaic virus* (TMV) infection [36]. In the current study, expression levels of acidic endochitinase Q from a significantly enriched MAPK signaling pathway was up-regulated over 48-fold following CWMV infection (Table 1), which indicated that the glyoxylate and dicarboxylate metabolism pathway played an important role in the response of *N. benthamiana* plants to CWMV infection. Plant RNA viral infections typically elicit leaf chlorosis, necrosis, plant stunting, and other symptoms, and leaf chlorosis frequently leads to decreased photosynthetic activity of the chloroplast [37, 38]. The chlorophyll a/b proteins (CAB) from the photosynthesis antenna protein pathway are membrane proteins that are vital for capturing light energy by merging with pigment molecules for photosynthesis [39]. In line with this result, we observed a few chlorotic lesions on leaves of plants inoculated with CWMV (Additional file 1: Fig. S1a). Furthermore, our results also showed that photosynthesis antenna protein pathway was significantly enriched. Several CAB proteins were down-regulated (Table 1), and more than half of the DEPs were predicted to be located in the chloroplast during CWMV infection (Fig. 2c), which suggests that chloroplasts functions may be impaired following CWMV invasion.

Numerous plant RNA viruses recruit the chloroplast membrane to facilitate infection and affect expression of many chloroplast-related genes including photosynthesis-related genes [32, 40, 41]. Chloroplasts are the source of numerous pro-defense signals, and they are closely related to the initiation of effector-triggered immunity [42–44]. More importantly, a series of key steps of the ABA biosynthesis pathway occurs in the chloroplast. The interactions of fungi and bacteria with ABA have been extensively studied; however, interactions between ABA and plant viruses are highly diverse and they are currently not well understood [45]. It is known that ABA synthesis pathway plays an important role during the plant viral infection. For instance, the expression levels of key ABA biosynthetic genes including *zeaxanthin epoxidase* (OsZEP1/OsABA1), *xanthoxin dehydrogenase* (OsABA2), 9-*cis*-*epoxycarotenoid dioxygenases* (OsNCED3) and *abscisic aldehyde oxidase* (OsAAO3) were decreased responsive to RBSDV infection [46]. *Bamboo mosaic virus* (BaMV) and *cucumber mosaic virus* (CWV) infection significantly enhanced the expression of several ABA biosynthetic genes including *ABA1*, *ABA2* and *AAO3* in *N. benthamiana* plants [47]. In the present study, label-free profiling combined with RT-qPCR analyses showed that mRNA and protein expression levels of ABA1 and ABA2 were significantly decreased in CWMV-infected plants, compared to those in the controls (Additional file 6: Table S4 and Fig. 6a). To investigate the relationships between the ABA pathway and CWMV in detail, RT-qPCR was performed, and the results showed that mRNA levels of ABA-biosynthetic genes including *NbNCED3* and *NbAAO3* were down-regulated responsive to CWMV infection (Fig. 7b). Moreover, we found that CWMV infection significantly decreased ABA concentrations (Fig. 7a). These results suggested that ABA biosynthetic pathway play an important part upon CWMV infection. It has been reported that there is tremendous feedback at every level of the ABA signaling network [48]. However, we observed that the expression levels of ABA-signal-transduction genes (*NbPYL6* and *NbPYL9*), and of ABA-responsive genes (*NbRAB18* and *NbABI5*) were also down-regulated in response to CWMV infection (Fig. 7b), which may be attributed to the cause that *PYL* genes function in multiple ways such as MAPK signaling and ABA signal transduction pathways [49]. And we had showed that MAPK signaling pathway also would be affected by CWMV infection (Table 1).

Viral infection typically induces significant changes in host transcript abundance of which a large number of transcription factors constitute an important part [50, 51]. We therefore hypothesized that CWMV infection would alter the expression of various transcription factors leading to reduced expression of ABA1 and ABA2 at both transcriptional and proteomic levels. Taken together, our results suggest that CWMV infection indeed suppressed the ABA pathway by interfering with ABA1 and ABA2 expression. Both ABA1 and ABA2 play important roles in the ABA biosynthesis pathway as ABA1 is responsible for catalyzing zeaxanthin to violaxanthin, and ABA2 is responsible for generating ABA aldehyde [52–54]. ABA1 and ABA2 have been reported to be strongly associated with BaMV accumulation in *A. thaliana* [47]. In the present study, we found that *ABA1* silencing significantly increased CWMV accumulation (Fig. 8f and g), and a similar pattern was observed after *ABA2* silencing (Fig. 8h and i). These results also suggest that NbABA1 and NbABA2 are positive regulators in response to CWMV infection. ABA played multiple parts in plant antiviral immune response. For instance, ABA treatment enhances resistance against TMV while a study on rice showed that ABA application increases susceptibility to RBSDV infection by suppressing the jasmonate pathway and increasing production of reactive oxygen species [46, 55]. In order to analyze the relationship between the ABA pathway and CWMV infection more comprehensively, ABA pre-treated plants were inoculated with CWMV and the results showed that ABA treatment alleviated CWMV infection in both *N. benthamiana* plants and wheat (Fig. 9 and Additional file 13: Fig. S7). These results together implied that ABA biosynthesis pathway played positive regulators in plant defense against CWMV infection.

## Conclusions

CWMV causes devastating damage to wheat production, and controlling this disease of winter wheat is diffcult [56, 57]. Breeding resistant wheat varieties is currently the most effective and economical countermeasure for preventing and controlling CWMV-induced disease; however, only few cultivars are resistant against CWMV [58]. In the current study, a proteomics approach was employed to explore proteomic changes in *N. benthamiana* during CWMV infection. A total of 390 DEPs was identified and characterized according to annotations. The photosynthesis antenna proteins, plant MAPK signaling, and the glyoxylate and dicarboxylate metabolism pathways were most significantly enriched regarding DEPs. CWMV infection suppressed the ABA pathway in *N. benthamiana*. In summary, our results also provide a foundation for identifying antiviral candidate factors to cultivate resistant varieties.

## Methods

### Plasmid construction

Plasmids pCB-35S-R1 and pCB-35S-R2 containing a full-length CWMV RNA1 or RNA2 sequence behind a 35S promotor were produced as previously described [31]. Partial sequences of the genes *NbABA1* (accession number XM_016620556) and *NbABA2* (accession number XM_016584441) were PCR-amplified using the primer pairs NbABA1-F^a^ with NbABA1-R^b^ and NbABA2-F^a^ with NbABA2-R^b^, respectively. PCR products were digested using the restriction enzymes *BamH*I *and Sma*I (New England Biolabs, Ipswich, MA, USA), and the products were individually cloned into TRV-based pTRV2 to generate pTRV2:NbABA1 and pTRV2:NbABA2 vectors. PCR products used for plasmid construction were generated using KOD DNA polymerase (TOYOBO, Kita-ku, Osaka, Japan). PCR primers used in this study are listed in Additional file 10: Table S5.

### VIGS

A TRV-based VIGS system in *N. benthamiana* was described as previously with small modifications [59]. pTRV2:NbABA1and pTRV2:NbABA2 vectors were individually transformed into *A. tumefaciens* strain GV3101 by electroporation. The agrobacterium cultures and *A. tumefaciens* strain GV3101 containing TRV RNA1 were grown overnight, pelleted, re-suspended in an induction buffer (1 M MgCl_2_, 10 mM MES, pH = 5.6, and 100 mM acetosyringone) and incubated for 3 h (h) at room temperature prior to leaf co-infiltration. Infiltrated leaves were collected at 7 days post agro-infiltration (dpai) and examined using RT-qPCR to confirm silencing of target-genes.

### Plant material and CWMV inoculation

The original *N. benthamiana* seeds were kindly donated by Pro. Yule Liu (Tsinghua University, China). *N. benthamiana* plants were grown in a greenhouse at 22 °C with a 16/8 h (light/dark) photoperiod until CWMV inoculation at the four-leaf stage. CWMV inoculation was performed as previously described with minor modifications [31]. The agrobacterium cultures carrying the recombinant binary constructs pCB-35S-R1 and pCB-35S-R2 were grown individually until approximately OD_600_ = 0.8. After centrifugation at 6000×g for 5 min, the supernatant was collected and re-suspended using an induction buffer (1 M MgCl_2_, 10 mM MES, pH 5.6, and 100 mM acetosyringone) for 3 h at room temperature. After this, the induction buffers containing pCB-35S-R1 or pCB-35S-R2 were mixed at equal volumes before leaf infiltration. All inoculated plants were grown in a constant-temperature incubator at 17 °C with a 14/10 h (light/dark) photoperiod. RT-qPCR and western blot were performed to confirm successful systemic infection at 14 dpi. Samples were collected for further analysis. Inoculation of wheat seedings with CWMV RNAs was performed as described previously [23]. In brief, plasmids pCB-35S-R1 carrying CWMV RNA1 and pCB-35S-R2 carrying CWMV RNA2 were linearized for vitro transcription. Vitro transcripts of CWMV RNA1 and RNA2 in a molar ratio of 1:1 were mixed with an equal amount of excess inoculation buffer (0.1 M glycine, 0.06 M potassium phosphate, 1% bentonite, 1% sodium pyrophosphate, 1% celite, pH 8.5) and then inoculated into leaves of wheat seedings.

### RNA extraction and RT-qPCR

Total RNA was isolated at 14 dpi using Trizol Reagent (Invitrogen, Carlsbad, CA, USA). First strand cDNA was synthesized using a First Strand cDNA Synthesis Kit (TOYOBO, Kita-ku, Osaka, Japan). The RT-qPCR reaction was performed using an ABI7900HT Sequence Detection System (Applied Biosystems, Foster City, CA, USA) with an AceQ qPCR SYBR Green Master Mix (Vazyme, China). Each treatment was performed using least three biological replicates with at least three technical replicates, each. Relative expression levels of ABA-related genes and CWMV CP were analyzed using the 2^-ΔΔC(t)^ method as described previously [60]. An actin gene was used as the internal reference for each reaction. These primers used in RT-qPCR are listed in Additional file 10: Table S5.

### Northern blot analysis

Northern blotting was conducted as described previously [6]. Briefly, 3 μg total RNA from each sample was loaded into a well of a 1.5% formaldehyde comprising agarose gel and separated by electrophoresis. Then, the separated RNAs were transferred onto Hybond-N+ membranes (Amersham Bioscience, Buckinghamshire, United Kingdom) and cross-linked for 2 h at 80 °C. CWMV genomic RNAs were analyzed with DIG-labeled DNA probes specific for the 3′-terminus of CWMV RNAs. The probe was made using a DIG High Prime DNA Labeling Kit II as instructed by the manufacturer (Roche, Basel, Switzerland). Finally, the blotting signal was detected with the Amersham Imager 600 (GE Healthcare Bio-Sciences, Pittsburgh, PA, USA).

### Western blotting

A western blot assay was performed as described previously with minor modifications [6, 31]. Samples were individually ground in liquid nitrogen and were then homogenized in a protein extraction buffer (Sigma-Aldrich, St. Louis, MO, USA) supplemented with Protease Inhibitor Cocktail Tablets (Roche, Basel, Switzerland; 1 tablet/50 mL buffer). After centrifugation at 16,000×g and 4 °C for 20 min, the supernatant was collected and was boiled for 10 min, after which proteins were separated using sodium dodecyl sulfate polyacrylamide gel electrophoresis before transfer to nitrocellulose membranes. A CWMV CP-specific antibody was produced in-house.

### Protein extraction

Protein extraction for nanoliquid chromatography (LC)-tandem MS (LC-MS/MS) was conducted as described previously with minor modifications [25]. Sample powder was collected and ground individually in liquid nitrogen. An amount of 0.1 g sample powder was transferred to extraction buffer (40 mM Tris-Cl, pH 8.5, 7 M urea, 2 M thiourea, 4%SDS, 1 mM PMSF, 10 mM DTT, and 2 mM EDTA), which was then vortexed thoroughly. The reaction was incubated on ice for 10 min. After centrifugation at 16,000×g and 4 °C for 20 min, the supernatant was mixed with the quadruple volume of cold acetone. The mixtures were incubated at − 20 °C overnight. After centrifugation at 16,000×g and 4 °C for 10 min, the supernatant was discarded, and the pellet was dried using a Speed-Vacuum concentrator. The dried pellet was dissolved using solution buffer containing 8 M urea and 100 mM triethylamonium bicarbonate (TEAB; pH 8.0) stored at − 80 °C. Protein concentration and quantification was assessed using an RC DC™ Protein Assay (Bio-Rad, Hercules, CA, USA).

### Protein digestion

The protein solution was mixed with 10 mM DTT for 30 min followed by incubation at 56 °C, after which 20 mM iodoacetamide was added, and the solutions were incubated at room temperature for 30 min. For trypsin digestion, protein samples were diluted five-fold using 100 mM TEAB. Trypsin was added at a ratio of 1:50 (mass ratio, trypsin: protein) for overnight digestion and at a ratio of 1:100 (mass ratio, trypsin: protein) for the second digestion step of 4 h. Approximately 150 μg sample was digested.

### LC-MS/MS analysis

LC-MS/MS analysis was performed as described previously with minor modifications [61, 62]. In brief, the reaction mixtures were dissolved using 0.1% formic acid and then loaded on an reversed-phase analytical column with 15 cm length and 75 μm i.d. A gradient of solvent contains 0.1% formic acid in 98% acetonitrile was produced from 6 to 23% for 25 min, from 23 to 35% within 8 min, then rising to 80% in 3 min, and remaining at 80% for the final 3 min, all at a constant flow rate of 400 nL/min on an EASY-nLC 1000 Ultrahigh Liquid Chromatography-triple Quadrupole Mass Spectrometry (UPLC) system. The peptides were subjected to an NSI source followed by MS/MS in Q Exactive™ Plus (Thermo Fisher Scientific, Waltham, MA, USA) together with UPLC. The electrospray voltage was set at 2.0 kV. Full scanning was conducted with the m/z scan ranging from 350 to 1800. Peptides were then picked out for MS/MS with NCE setting at 28, and fragments were detected in the Orbitrap at a resolution of 17,500. A data-dependent procedure alternated between one MS scan and 20 MS/MS scans with 15 s dynamic exclusion. Automatic gain control was set to 5E4. Fixed first mass was set to 100 m/z.

### Database search

Database search was conducted as previously described with minor modifications [61, 62]. The Maxquant search engine (v.1.5.2.8) was used to process the resulting MS/MS data. Then the tandem mass spectra were searched against the UniProt *Nicotiana tabacum* database (73,605 sequences; updated in May 2019). In the first and main search precursor mass and fragment mass had an initial mass tolerance of 20 ppm and 5 ppm, respectively. The search included variable modifications of methionine oxidation, and fixed modification of carbamidomethyl cysteine. Trypsin/P was specified as cleavage enzyme and a maximum of two miscleavages was allowed. The false discovery rate (FDR) was set to 0.01 for peptide and protein identifications. Protein quantification was performed with MaxQuant as previously described with minor modifications [63]. *P*-values were corrected according to the FDR and were applied for statistical analyses to estimate differences between infected and control samples. Intensity-based absolute quantification in MaxQuant was performed on the identified peptides to quantify protein abundance. Differentially expressed proteins were filtered at a fold change of > 1.5 and an FDR *P*-value < 0.05.

### Protein annotation

GO annotation was produced for protein sequences obtained from the UniProt-GOA database (www.http://www.ebi.ac.uk/GOA/). First, the ID of the identified protein was converted to a UniProt ID and was then mapped to GO IDs by protein ID. For identified proteins that were not annotated in the UniProt-GOA database, InterProScan software (v.5.14–53.0) (www.http://www.ebi.ac.uk/interpro) was used to annotate each protein’s GO function. Then, the proteins were classified by GO annotation according to three groups: biological processes, cellular components, and molecular functions.

Proteins in eukaryotic cells are localized in various cellular organelles, depending on what membrane structure they bind to. The main subcellular localization of eukaryotic cells includes the extracellular space, cytoplasm, the nucleus, mitochondria, peroxisomes, vacuoles, the Golgi apparatus, the endoplasmic reticulum, the cytoskeleton, nucleoplasm, the nuclear matrix, and the ribosome. We used Wolfpsort (v.0.2) (www.http://www.genscript.com/psort/wolf_psort.html), a subcellular localization predication software. Wolfpsort is an updated version of PSORT/PSORT II, which is used for predicting eukaryotic sequences.

The KEGG database was used for pathway annotation. First, the KEGG online tool KAAS was used to annotate the KEGG database description of proteins, after which the annotation result was mapped using the KEGG pathway database and the KEGG online tool KEGG mapper.

The domain functional descriptions of identified proteins were annotated using InterProScan (a sequence analysis application) based on a protein sequence alignment method, and the InterPro domain database was used. InterPro (http://www.ebi.ac.uk/interpro/) is a database that integrates diverse information on protein families, domains, and functional sites and makes it freely available to the public via web-based interfaces and services. Central to the database are diagnostic models, known as signatures, against which protein sequences can be searched to determine their potential function. InterPro can be utilized in large-scale analysis of whole genomes and meta-genomes as well as for characterizing individual protein sequences.

### Functional enrichment analysis

Proteins were classified by GO annotation into three categories: biological processes, cellular compartments, and molecular functions. For each category, a two-tailed Fisher’s exact test was employed to test enrichment of DEPs against all identified proteins. The GO with a *P* value < 0.05 and false discovery rate <1% was considered significant.

The KEGG database was used to identify enriched pathways using a two-tailed Fisher’s exact test to test enrichment of DEPs against all identified proteins. Pathways with a corrected *P* value < 0.05 and false discovery rate <1% were considered significant. These pathways were classified into hierarchical categories according to the KEGG website.

For each category of proteins, InterPro (a resource that provides functional analysis of protein sequences by classifying them into families and predicting the presence of domains and important sites) database was searched, and a two-tailed Fisher’s exact test was employed to test enrichment of DEPs against all identified proteins. Protein domains with a *P* value < 0.05 were considered significant.

### ABA and ABA inhibitor treatments (NDGA)

ABA (Sigma-Aldrich, St. Louis, MO, USA) was dissolved in 0.2% ethanol to a final concentration of 100 μM. NDGA (Sigma-Aldrich, St. Louis, MO, USA), targeting 9-*cis*-epoxycarotenoid dioxygenase, was dissolved using 0.2% ethanol to 10 mM. *N. benthamiana* plants were treated with 100 μM ABA solution, 10 mM NDGA solution, or 0.2% ethanol solution (control). Treatment solutions were applied on the adaxial and abaxial sides of leaves until solution drops dripped off the leaves. After 12 h, pre-treated *N. benthamiana* leaves were inoculated with CWMV.

### Analysis of ABA contents

Samples were collected from experimental plants for ABA extraction as described previously [64]. Samples were then ground in liquid nitrogen and were mixed individually (200 mg leaf powder per sample) with ^2^H_5_-ABA (45 pmol). Two milliliters of methanol were added to each sample, which was then mixed, and the mixture was incubated overnight at − 20 °C. After centrifugation at 160,000×g and 4 °C for 20 min, the supernatant was collected and dried under nitrogen gas. The pellet was dissolved in 1 mL 5% ammonia solution and purified using Oasis MAX SPE columns (Waters, Milford, MA, USA) as the manufacturer’s instructions. Eluted ABA was dried under nitrogen gas, dissolved using 200 μL water/methanol mixture (20:80, v/v), and was then analyzed by Ultrahigh Liquid Chromatography-triple Quadrupole Mass Spectrometry (UPLC-MS/MS). Three independent biological replicates were used.

## Supplementary Information

**Additional file 1: Figure S1.** CWMV-infected *N. benthamiana* plants. **a** Morphological comparison between control and CWMV-infected plants. **b** PCR assay for detecting CWMV *CP* and *MP* gene. Lanes 1 to 3 below mock, samples were prepared from mock plants. Lanes 1 to 3 below CWMV-infected, samples were prepared from *N. benthamiana* by 14 dpi. PC, positive control. NC, negative control. **c** Western blot assay for detecting CWMV CP. Lanes 1 to 3 below mock, samples were prepared from controls. Lanes 1 to 3 below CWMV-infected, samples were prepared from *N. benthamiana* by 14 dpi. Coomassie brilliant blue-stained loadings are shown in the lower section of the figure. Controls were agro-infiltrated with the agrobacterium cultures carrying the empty vector pCB-35S.

**Additional file 2: Table S1.** Detailed information of identified peptides.

**Additional file 3: Table S2.** Detailed information of all identified proteins.

**Additional file 4: Table S3.** Detailed information of all DEPs.

**Additional file 5: Figure S2.** Numbers of up-regulated and down- regulated DEPs in CWMV-infected plants compared to that in control plants. Controls were agro-infiltrated with the agrobacterium cultures carrying the empty vector pCB-35S.

**Additional file 6: Table S4.** Basic information of selected DEPs for RT-qPCR validation.

**Additional file 7: Figure S3.** Subcellular location of up-regulated and down-regulated DEPs. **a** Subcellular location of up-regulated DEPs. **b** Subcellular location of down-regulated DEPs.

**Additional file 8: Figure S4.** Multiple sequence alignment result. Amino acid sequence of Polypeptide (NbABA1) was aligned with sequences of *N. tabacum*, and *A. thaliana* ABA1 sequences using DNAMAN software.

**Additional file 9: Figure S5.** Multiple sequence alignment result. Amino acid sequence of Polypeptide (NbABA2) was aligned with sequences of *N. tabacum*, and *A. thaliana* ABA2 sequences using DNAMAN software.

**Additional file 10: Table S5.** Primers used in this study.

**Additional file 11.** Full length image of Figure S1b, Figure S1c, Fig. 8g, Fig. 8i, Fig. 9b, Fig. 9c and Fig. 9d.

**Additional file 12: Figure S6.** Effects of applying ABA on CWMV infection in wheat. RT-qPCR showing mRNA expression of CWMV *CP*. Samples were collected from the systemic leaves of pre-treated CWMV-inoculated wheat. Means ± SE were calculated from three biological replicates relative to plants, and each replicate comprised three technical replicates. **, *P* < 0.01 (Student’s *t*-test).

**Additional file 13: Figure S7.** Effects of ABA application on CWMV RNA2 inoculated plants. RT-qPCR showing mRNA expression of CWMV RNA2 *CP* and *CRP*. Samples were collected from inoculated leaves 3 days after agrobacterium infiltration.

## Data Availability

All data generated or analyzed in this study are included in this article and its supplementary materials. All raw mass spectrometry (MS) data files has been deposited and can be access on the proteomeXchange with the dataset identifier PXD017593 (https://www.ebi.ac.uk/pride/profile/hnndhelong2).

## Abbreviations

CWMV: *Chinese wheat mosaic virus*
*P. graminis*: *Polymyxa graminis*
MS: mass spectrometry
DEPs: differentially expressed proteins
*N. benthamiana*: *Nicotiana benthamiana*
ABA: abscisic acid
ABA1: zeaxanthin epoxidase
ABA2: xanthoxin dehydrogenase

## References

[1] Sanfaçon H (2017). Grand challenge in plant virology: understanding the impact of plant viruses in model plants, in agricultural crops, and in complex ecosystems. Front Microbiol.

[2] Alexander HM (2014). Plant-virus interactions and the agro-ecological interface. Eur J Plant Pathol.

[3] Wang A (2015). Dissecting the molecular network of virus-plant interactions: the complex roles of host factors. Annu Rev Phytopathol.

[4] Garciaruiz H (2019). Host factors against plant viruses. Mol Plant Pathol.

[5] Lian J (2016). *Rice Dwarf Virus* P2 protein hijacks auxin signaling by directly targeting the rice OsIAA10 protein, enhancing viral infection and disease development. PLoS Pathog.

[6] He L (2020). *Rice Black-Streaked Dwarf Virus* encoded P5-1 regulates the ubiquitination activity of SCF E3 ligases and inhibits jasmonate dignaling to benefit its infection in rice. New Phytol.

[7] Meena RP (2013). Hydro-priming of seed improves the water use efficiency, grain yield and net economic return of wheat under different moisture regimes. J Integr Agr..

[8] Foods FS (2017). What do people eat.

[9] Guo L (2019). *Chinese wheat mosaic virus*: a long-term threat to wheat in China. J Integr Agr.

[10] King AM, et al. Virus taxonomy: ninth report of the international committee on taxonomy of viruses, vol. 9: Elsevier; 2011.

[11] Ye Y, Gong Z (1998). Cloning, expression and identification of matrix protein gene of wheat rosette stunt virus. Acta Bioch Bioph Sin.

[12] Kanyuka K, Ward E, Adams MJ (2003). *Polymyxa graminis* and the cereal viruses it transmits: a research challenge. Mol Plant Pathol.

[13] Chen JP (1993). Occurrence of fungally transmitted wheat mosaic viruses in China. Ann Appl Biol..

[14] Adams MJ, Kreuze J (2009). A new family of rod-shaped plant viruses. Arch Virol.

[15] Diao A (1999). Complete sequence and genome properties of *Chinese wheat mosaic virus*, a new furovirus from China. J Gen Virol..

[16] Ye R (1999). Characterisation and partial sequence of a new *furovirus* of wheat in China. Plant Pathol.

[17] Yang J (2001). Sequence of a second isolate of Chinese wheat mosaic *furovirus*. J Phytopathol.

[18] Andika IB (2013). Endoplasmic reticulum export and vesicle formation of the movement protein of *Chinese wheat mosaic virus* are regulated by two transmembrane domains and depend on the secretory pathway. Virology..

[19] Sun L (2013). The CUG-initiated larger form coat protein of *Chinese wheat mosaic virus* binds to the cysteine-rich RNA silencing suppressor. Virus Res.

[20] Sun L (2013). Identification of the amino acid residues and domains in the cysteine-rich protein of *Chinese wheat mosaic virus* that are important for RNA silencing suppression and subcellular localization. Mol Plant Pathol.

[21] Andika IB (2013). Root-specific role for *Nicotiana benthamiana* RDR6 in the inhibition of *Chinese wheat mosaic virus* accumulation at higher temperatures. Mol Plant-Microbe In.

[22] Yang J (2017). A furoviral replicase recruits host HSP70 to membranes for viral RNA replication. Sci Rep.

[23] Yang J (2020). *Chinese wheat mosaic virus*-derived vsiRNA-20 can regulate virus infection in wheat through inhibition of vacuolar-(H^+^)-PPase induced cell death. New Phytol.

[24] Fu S (2017). *Rice Stripe Virus* interferes with S-acylation of Remorin and induces its Autophagic degradation to facilitate virus infection. Mol Plant.

[25] Yue R (2018). Comparative proteomic analysis of maize (*Zea mays* L.) seedlings under r*ice black-streaked dwarf virus* infection. BMC Plant Biol.

[26] Dang M (2019). Proteomic changes during MCMV infection revealed by iTRAQ quantitative proteomic analysis in maize. In J Mol Sci.

[27] Bombarely A (2012). A draft genome sequence of *Nicotiana benthamiana* to enhance molecular plant-microbe biology research. Mol Plant Microbe In..

[28] Jiang S (2014). Heat shock protein 70 is necessary for *rice stripe virus* infection in plants. Mol Plant Pathol.

[29] Yang M (2018). *Barley stripe mosaic virus* γb protein subverts autophagy to promote viral infection by disrupting the ATG7-ATG8 interaction. Plant Cell.

[30] Goodin M (2008). *Nicotiana benthamiana*: its history and future as a model for plant–pathogen interactions. Mol Plant Microbe In..

[31] Yang J (2016). Functional identification of two minor capsid proteins from *Chinese wheat mosaic virus* using its infectious full-length cDNA clones. J Gen Virol.

[32] Mochizuki T, Ogata Y, Ohki ST (2014). Quantitative transcriptional changes associated with chlorosis severity in mosaic leaves of tobacco plants infected with *cucumber mosaic virus*. Mol Plant Pathol.

[33] Chen T (2016). iTRAQ-based quantitative proteomic analysis of cotton roots and leaves reveals pathways associated with salt stress. PloS one..

[34] Zhao X (2020). iTRAQ-based quantitative proteomic analysis of the response of *Hylotelephium erythrostictum* leaves to salt stress. Sci Hortice.

[35] Meng X, Zhang S (2013). MAPK cascades in plant disease resistance signaling. Annu Rev Phytopathol.

[36] Payne G (1990). Isolation of complementary DNA clones encoding pathogenesis-related proteins P and Q, two acidic chitinases from tobacco. P Natl Acad Sci USA.

[37] Zhao J (2019). Characterization of proteins involved in chloroplast targeting disturbed by rice stripe virus by novel protoplast–chloroplast proteomics. Int J Mol Sci.

[38] Bhor SA (2017). Inducible transgenic tobacco system to study the mechanisms underlying chlorosis mediated by the silencing of chloroplast heat shock protein 90. Virus Disease.

[39] Green BR, Durnford DG (1996). The chlorophyll-carotenoid proteins of oxygenic photosynthesis. Annu Rev Plant Physiol.

[40] Postnikova OA, Nemchinov LG (2012). Comparative analysis of microarray data in *Arabidopsis* transcriptome during compatible interactions with plant viruses. Virol J.

[41] Wei T (2010). Sequential recruitment of the endoplasmic reticulum and chloroplasts for plant potyvirus replication. J Virol.

[42] Alazem M, Lin NS (2015). Roles of plant hormones in the regulation of host–virus interactions. Mol Plant Pathol.

[43] Ding X (2019). Chloroplast clustering around the nucleus is a general response to pathogen perception in *Nicotiana benthamiana*. Mol Plant Pathol.

[44] Bhattacharyya D, Chakraborty S (2018). Chloroplast: the Trojan horse in plant–virus interaction. Mol Plant Pathol.

[45] Alazem M, Lin NS (2017). Antiviral roles of abscisic acid in plants. Front Plant Sci.

[46] Xie K (2018). Abscisic acid negatively modulates plant defence against *rice black-streaked dwarf virus* infection by suppressing the jasmonate pathway and regulating reactive oxygen species levels in rice. Plant Cell Environ.

[47] Alazem M, Lin K, Lin N (2014). The abscisic acid pathway has multifaceted effects on the accumulation of *bamboo mosaic virus*. Mol Plant Microbe In.

[48] Sean RC (2010). Abscisic acid: emergence of a core signaling network. Annul Rev Plant Biol.

[49] Axel DZ (2016). The role of MAPK modules and ABA during abiotic stress signaling. Trends Plant Sci.

[50] Rodrigo G (2012). A meta-analysis reveals the commonalities and differences in *Arabidopsis thaliana* response to different viral pathogens. PLoS One.

[51] Rianopachon DM (2007). An integrative plant transcription factor database. BMC Bioinformatics.

[52] Marin E (1996). Molecular identification of zeaxanthin epoxidase of *Nicotiana plumbaginifolia*, a gene involved in abscisic acid biosynthesis and corresponding to the ABA locus of *Arabidopsis thaliana*. EMBO J.

[53] Audran C (1998). Expression studies of the zeaxanthin epoxidase gene in *Nicotiana plumbaginifolia*. Plant Physiol.

[54] González M (2002). The short-chain alcohol dehydrogenase ABA2 catalyzes the conversion of xanthoxin to abscisic aldehyde. Plant Cell.

[55] Whenham R (1986). *Tobacco mosaic virus* induced increase in abscisic-acid concentration in tobacco leaves. Planta..

[56] Adams M (1991). Transmission of plant viruses by fungi. Ann Appl Biol.

[57] Shirako Y, Suzuki N, French RC (2000). Similarity and divergence among viruses in the genus *Furovirus*. Virology..

[58] Yang J (2002). Responses of some American, European and Japanese wheat cultivars to soil-borne wheat viruses in China. Agr Sci China.

[59] Liu Y, Schiff M, Dinesh-Kumar S (2002). Virus-induced gene silencing in tomato. Plant J.

[60] Livak K, Schmittgen T. Analysis of relative gene expression data using real-time quantitative PCR and the 2^-△△Ct^ method. Methods. 2000; 25(4)402-8. 10.1006/meth.2001.1262 11846609

[61] Li W (2018). Integrative analysis of proteome and ubiquitylome reveals unique features of lysosomal and endocytic pathways in gefitinib resistant non-small cell lung cancer cells. Proteomics..

[62] Wang JH, et al. Protein modification characteristics of the malaria parasite *Plasmodium falciparum* and the infected erythrocytes. Mol Cell Proteomics. 2020 Doi: 10.1074/mcp.RA120.002375). 10.1074/mcp.RA120.002375 PMC7857547 33517144

[63] Soares EDA (2017). Label-free quantitative proteomic analysis of pre-flowering PMeV-infected *Carica papaya L*. J Proteome.

[64] Fu J (2012). Simple, rapid, and simultaneous assay of multiple carboxyl containing phytohormones in wounded tomatoes by UPLC-MS/MS using single SPE purification and isotope dilution. Anal Sci.

