# Supplementary figures and images for: Comparative proteomic analysis of Nicotiana benthamiana plants under Chinese wheat mosaic virus infection
Source: BMC Plant Biol. 2021 Jan 19;21:51. doi: 10.1186/s12870-021-02826-9 (PMC7816467; doi:10.1186/s12870-021-02826-9)

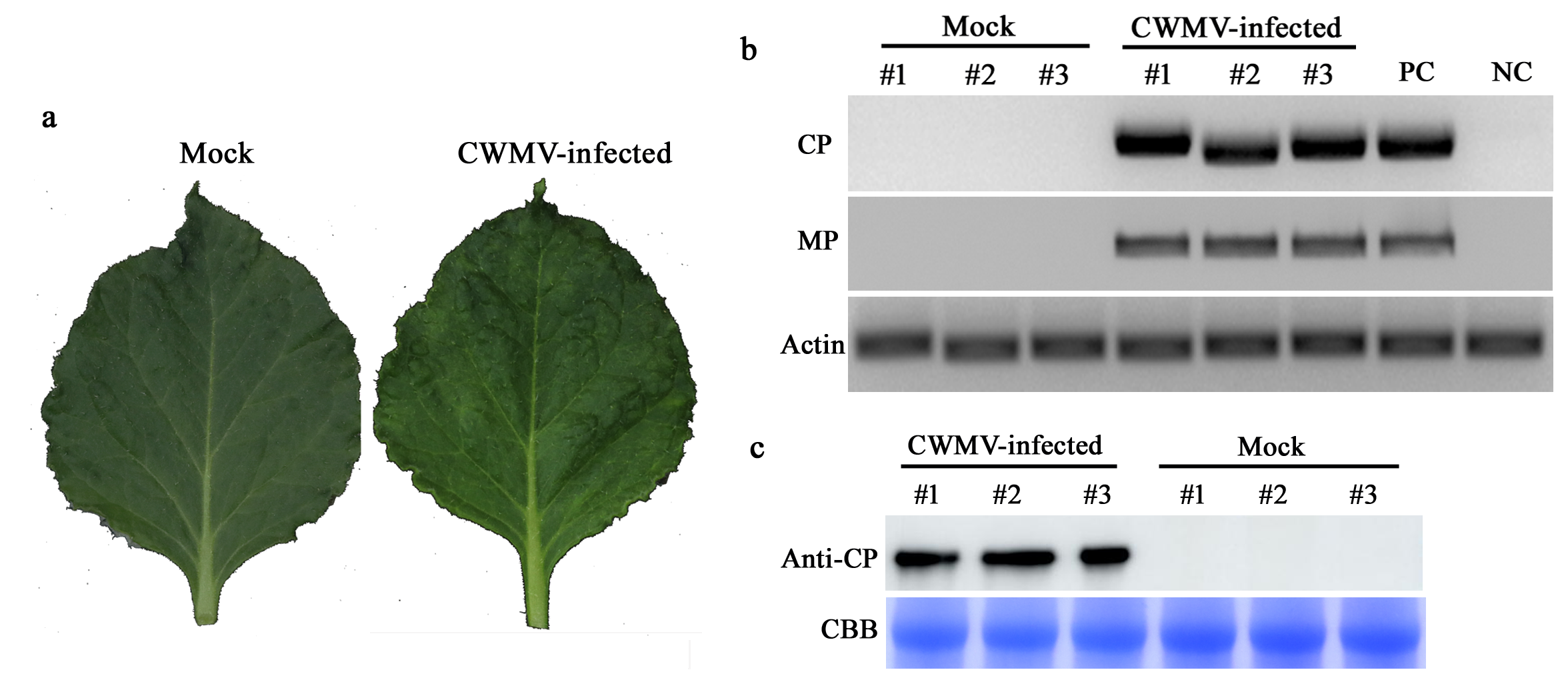

Supplement: Supplementary file 1 — Additional file 1: Figure S1. CWMV-infected N. benthamiana plants. a Morphological comparison between control and CWMV-infected plants. b PCR assay for detecting CWMV CP and MP gene. Lanes 1 to 3 below mock, samples were prepared from mock plants. Lanes 1 to 3 below CWMV-infected, samples were prepared from N. benthamiana by 14 dpi. PC, positive control. NC, negative control. c Western blot assay for detecting CWMV CP. Lanes 1 to 3 below mock, samples were prepared from controls. Lanes 1 to 3 below CWMV-infected, samples were prepared from N. benthamiana by 14 dpi. Coomassie brilliant blue-stained loadings are shown in the lower section of the figure. Controls were agro-infiltrated with the agrobacterium cultures carrying the empty vector pCB-35S. [file 12870_2021_2826_MOESM1_ESM.tif]

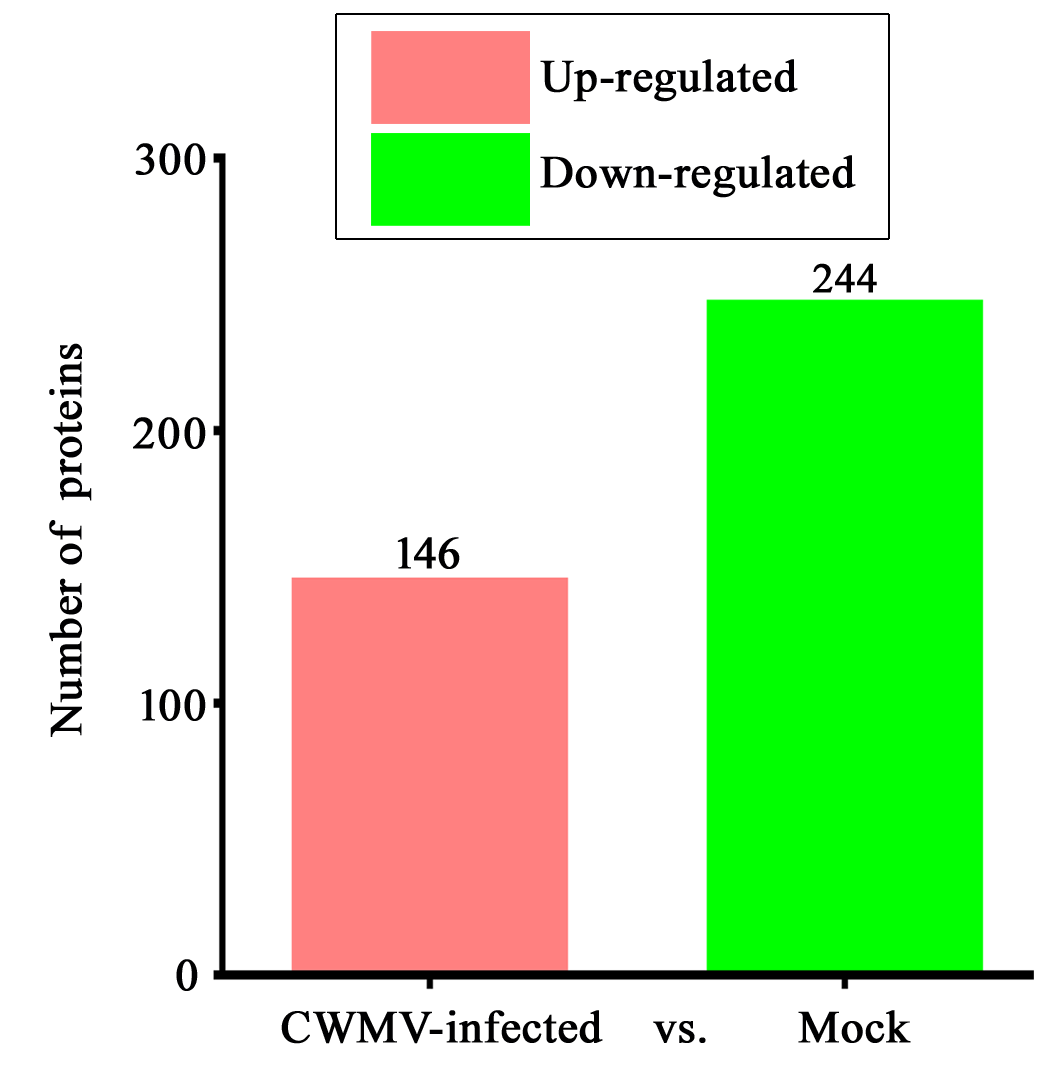

Supplement: Supplementary file 5 — Additional file 5: Figure S2. Numbers of up-regulated and down- regulated DEPs in CWMV-infected plants compared to that in control plants. Controls were agro-infiltrated with the agrobacterium cultures carrying the empty vector pCB-35S. [file 12870_2021_2826_MOESM5_ESM.tif]

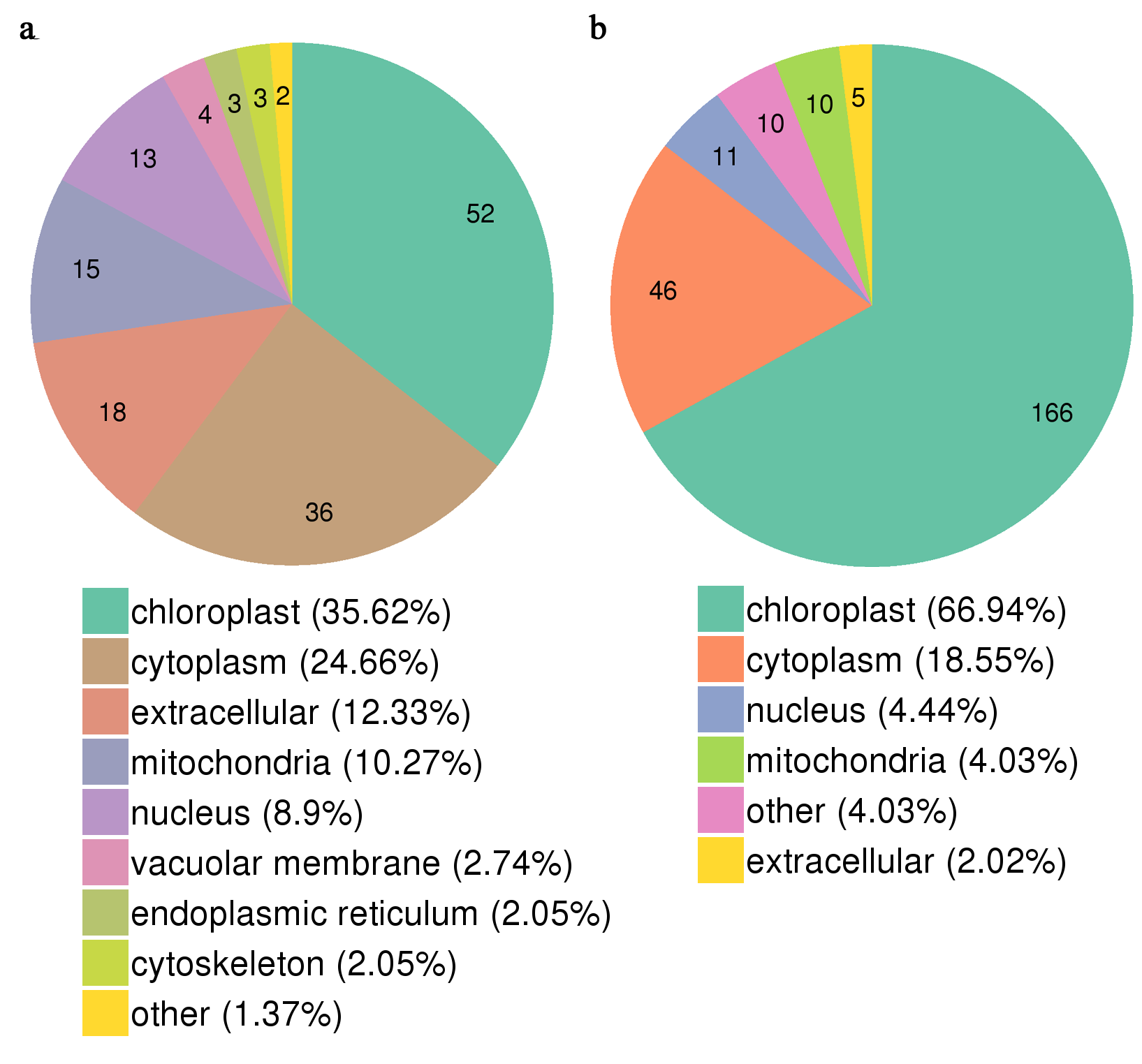

Supplement: Supplementary file 7 — Additional file 7: Figure S3. Subcellular location of up-regulated and down-regulated DEPs. a Subcellular location of up-regulated DEPs. b Subcellular location of down-regulated DEPs. [file 12870_2021_2826_MOESM7_ESM.tif]

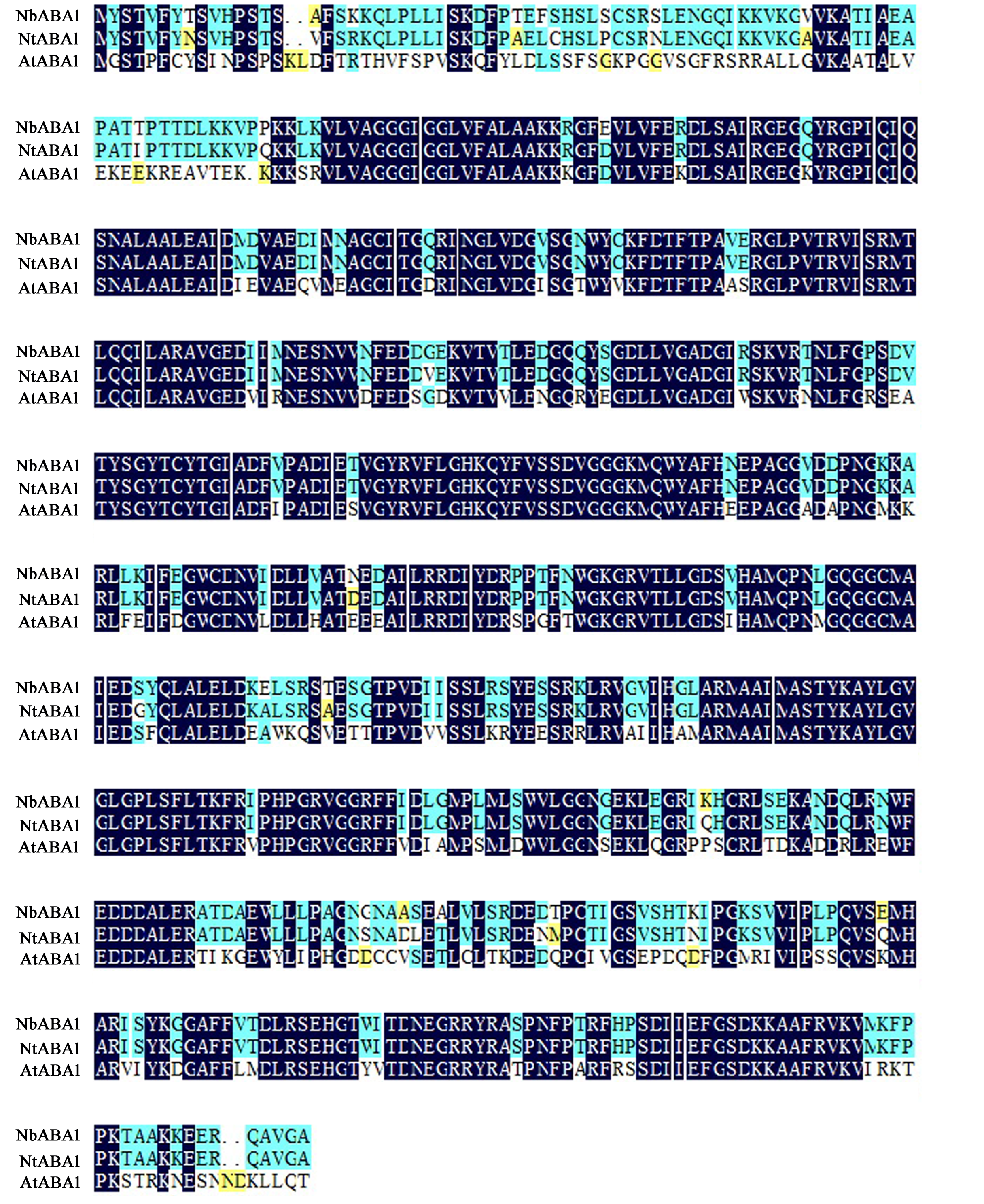

Supplement: Supplementary file 8 — Additional file 8: Figure S4. Multiple sequence alignment result. Amino acid sequence of Polypeptide (NbABA1) was aligned with sequences of N. tabacum, and A. thaliana ABA1 sequences using DNAMAN software. [file 12870_2021_2826_MOESM8_ESM.tif]

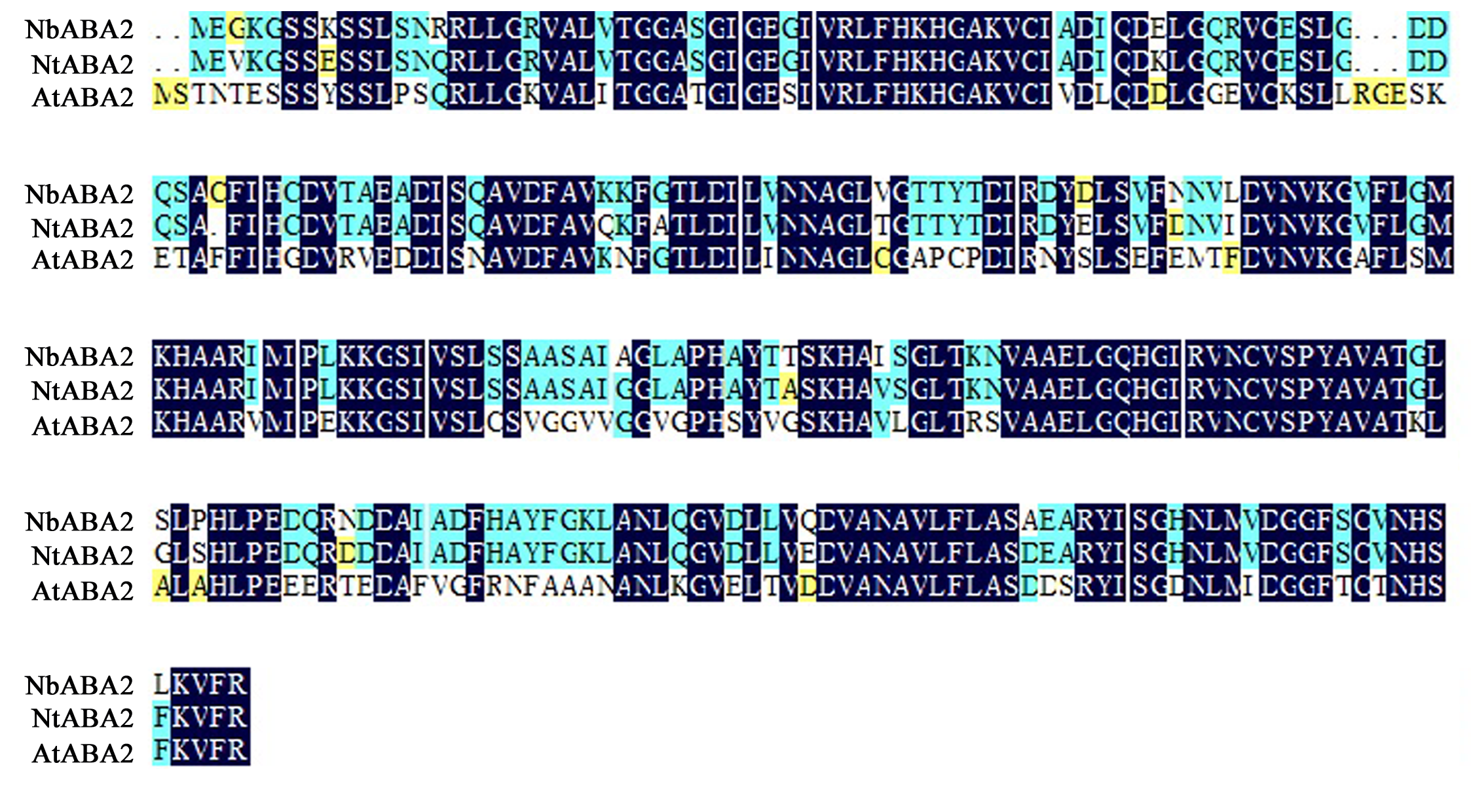

Supplement: Supplementary file 9 — Additional file 9: Figure S5. Multiple sequence alignment result. Amino acid sequence of Polypeptide (NbABA2) was aligned with sequences of N. tabacum, and A. thaliana ABA2 sequences using DNAMAN software. [file 12870_2021_2826_MOESM9_ESM.tif]

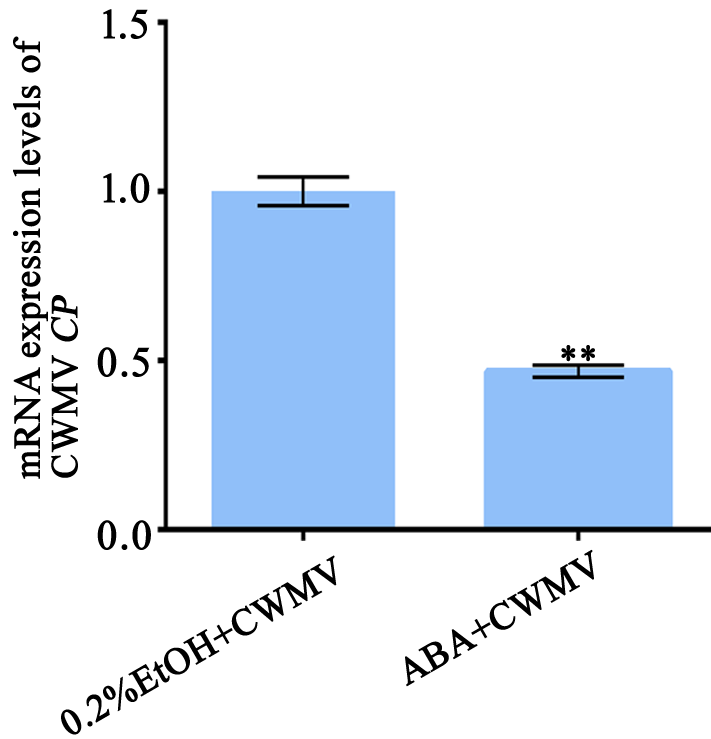

Supplement: Supplementary file 12 — Additional file 12: Figure S6. Effects of applying ABA on CWMV infection in wheat. RT-qPCR showing mRNA expression of CWMV CP. Samples were collected from the systemic leaves of pre-treated CWMV-inoculated wheat. Means ± SE were calculated from three biological replicates relative to plants, and each replicate comprised three technical replicates. **, P < 0.01 (Student’s t-test). [file 12870_2021_2826_MOESM12_ESM.tif]

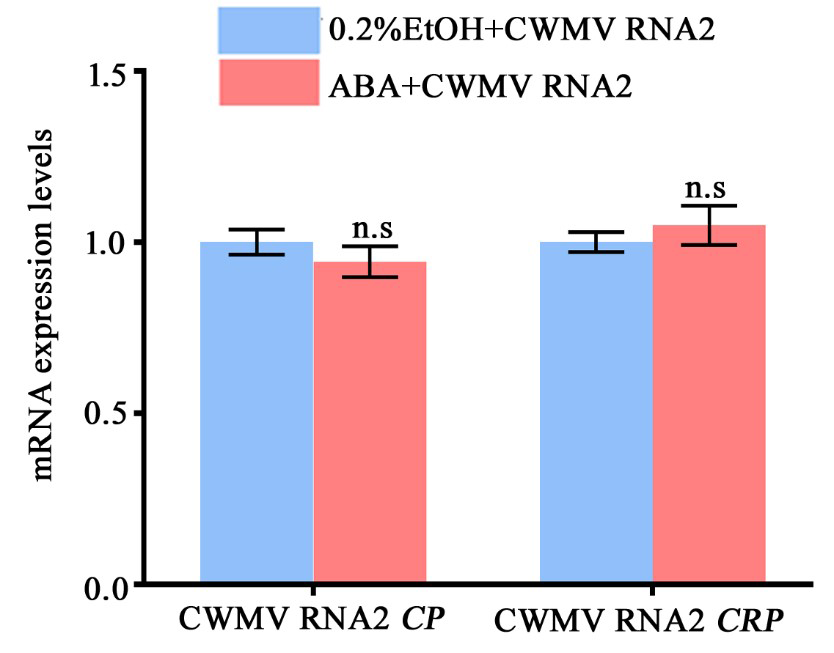

Supplement: Supplementary file 13 — Additional file 13: Figure S7. Effects of ABA application on CWMV RNA2 inoculated plants. RT-qPCR showing mRNA expression of CWMV RNA2 CP and CRP. Samples were collected from inoculated leaves 3 days after agrobacterium infiltration. [file 12870_2021_2826_MOESM13_ESM.tif]
