# Supplementary material for: Comparative proteomic analysis of Nicotiana benthamiana plants under Chinese wheat mosaic virus infection
Source: BMC Plant Biol. 2021 Jan 19;21:51. doi: 10.1186/s12870-021-02826-9 (PMC7816467; doi:10.1186/s12870-021-02826-9)
Supplement: Supplementary file 11 — Additional file 11. Full length image of Figure S1b, Figure S1c, Fig. 8g, Fig. 8i, Fig. 9b, Fig. 9c and Fig. 9d. [file 12870_2021_2826_MOESM11_ESM.docx]

**Figure S1b**


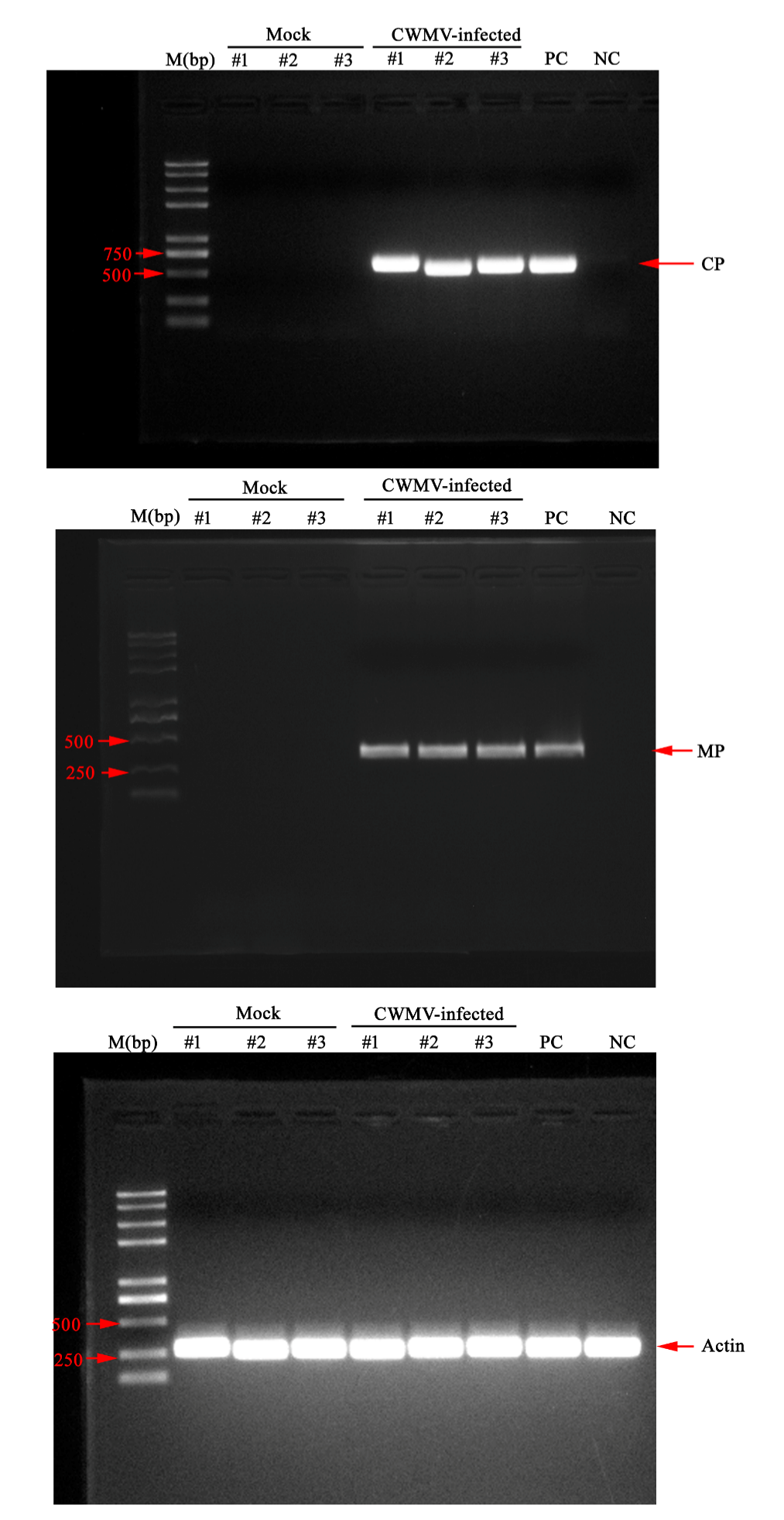


**Figure S1b** RT-PCR detection of CWMV *CP* and *MP*. Lanes 1 to 3 below mock, samples were prepared from mock plants. Lanes1 to 3 below CWMV-infected, samples were prepared from *N. benthamiana* by 14 d post CWMV inoculation (dpi). PC, positive control. NC, negative control.

**Figure S1c**


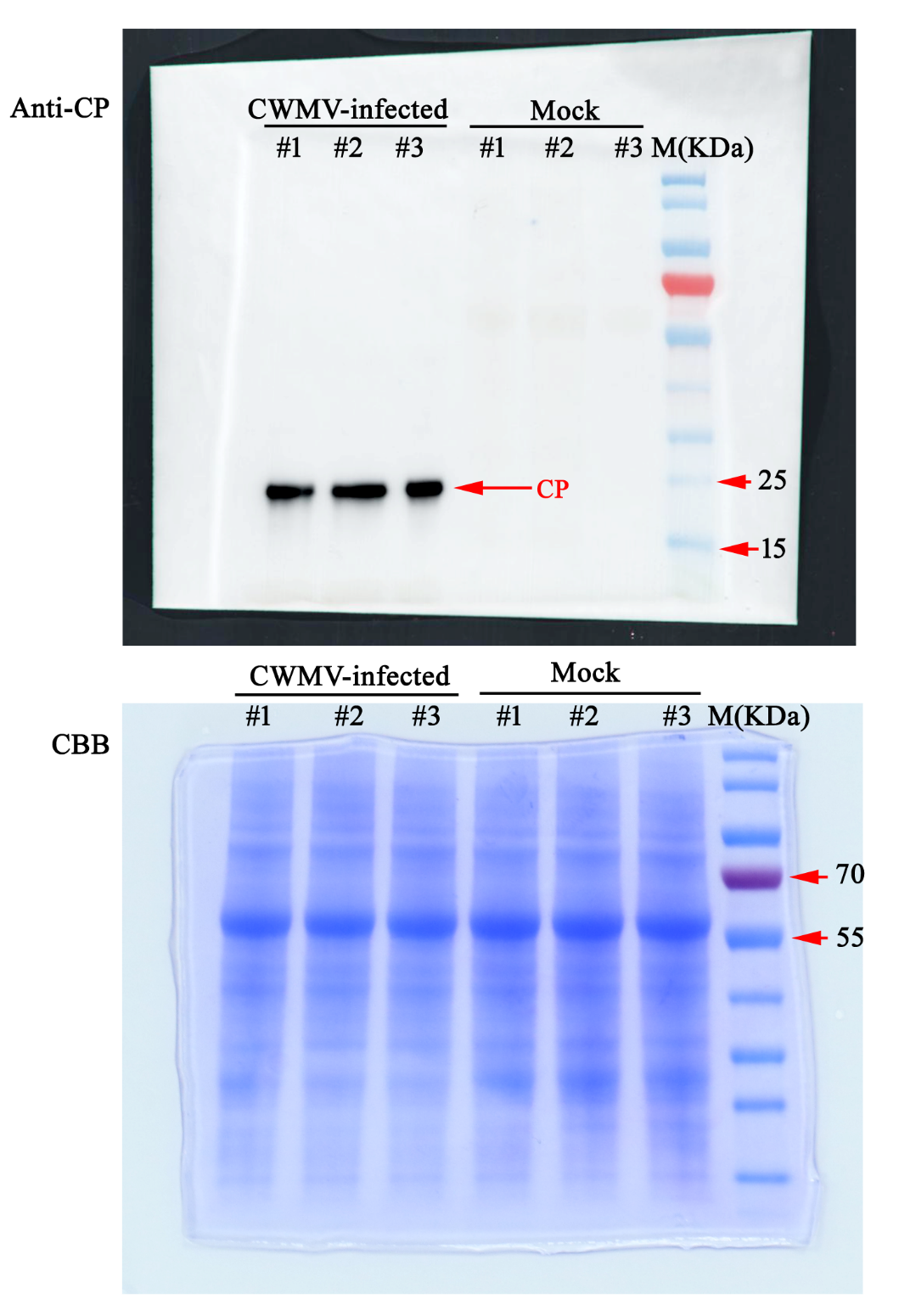


**Figure S1c** Western blot assay for detecting CWMV CP protein. Lanes 1 to 3 below mock, samples were prepared from mock plants. Lanes 1 to 3 below CWMV-infected, samples were prepared from *N. benthamiana* by 14 d post CWMV inoculation (dpi). Coomassie brilliant blue (CBB)-stained loadings are shown in the bottom of the figure.

**Figure 8g**


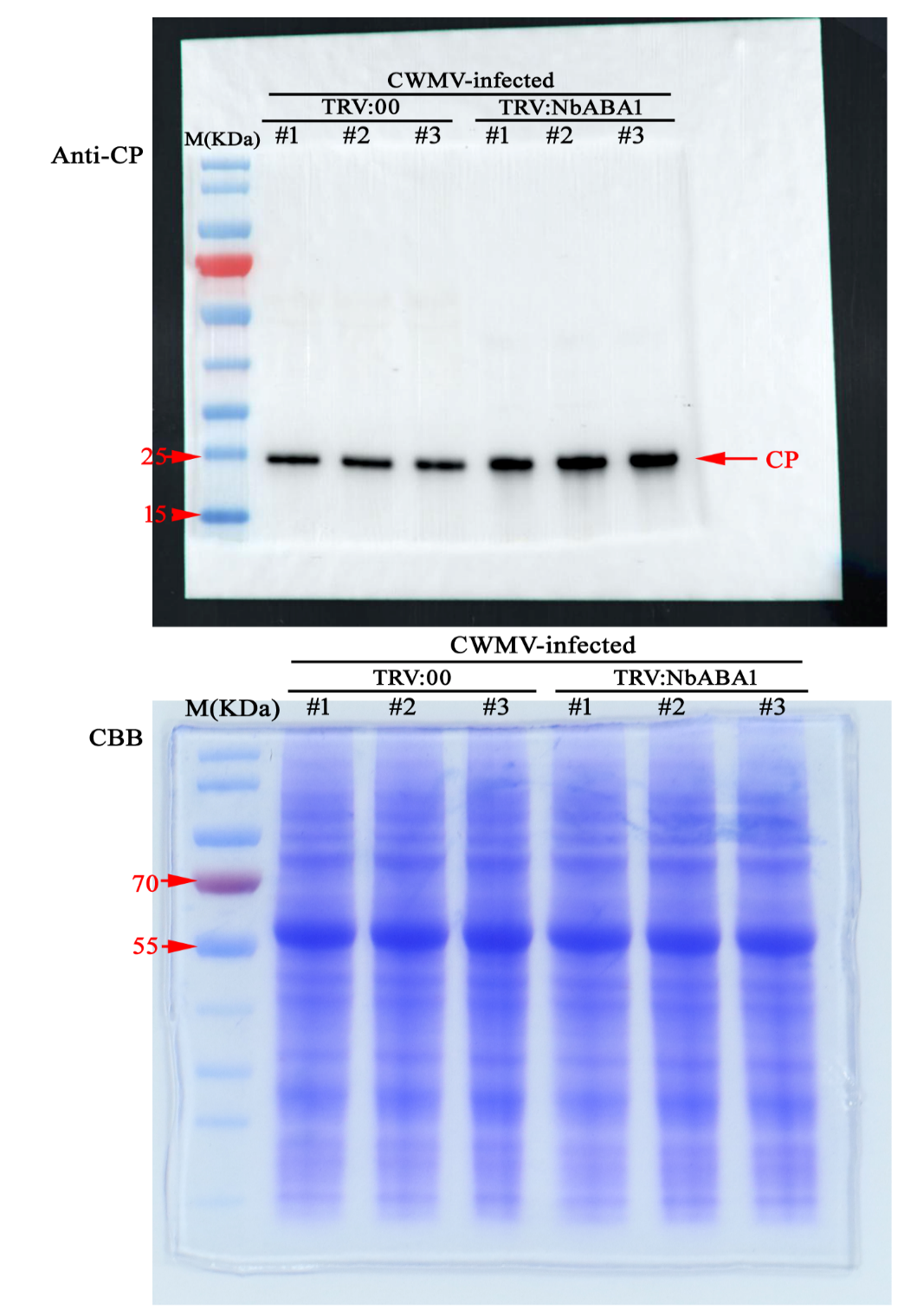


**Figure 8g** **right** Western blot assay showing protein expression of CWMV CP in *NbABA1*-slienced plants inoculated with CWMV. Coomassie brilliant blue (CBB)-stained loadings are shown in the bottom of the figure.

**Figure 8i**


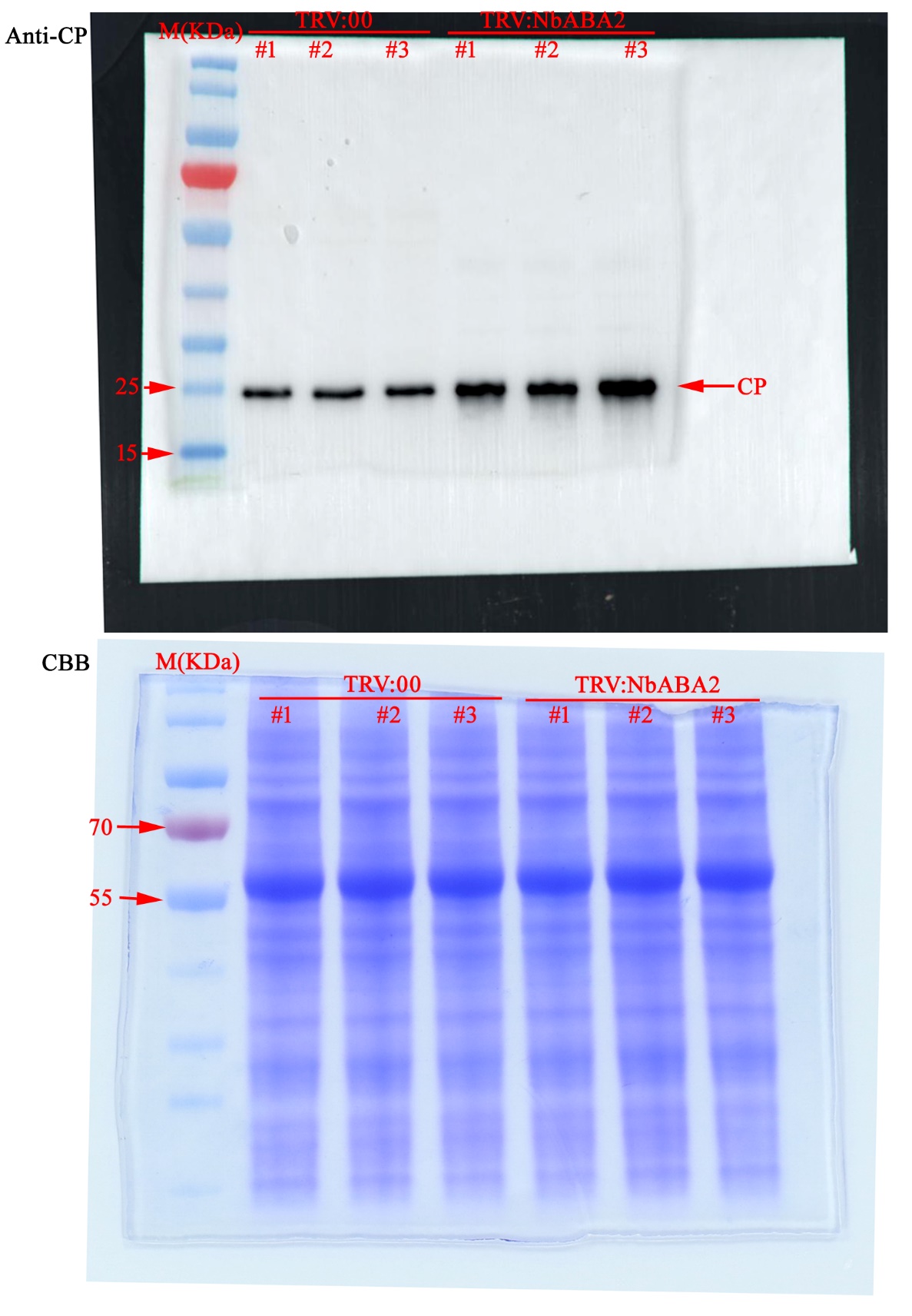


**Figure 8i** Western blot assay showing protein expression of CWMV CP in *NbABA2*-slienced plants inoculated with CWMV. Coomassie brilliant blue (CBB)-stained loadings are shown in the bottom of the figure.

**Figure 9b**


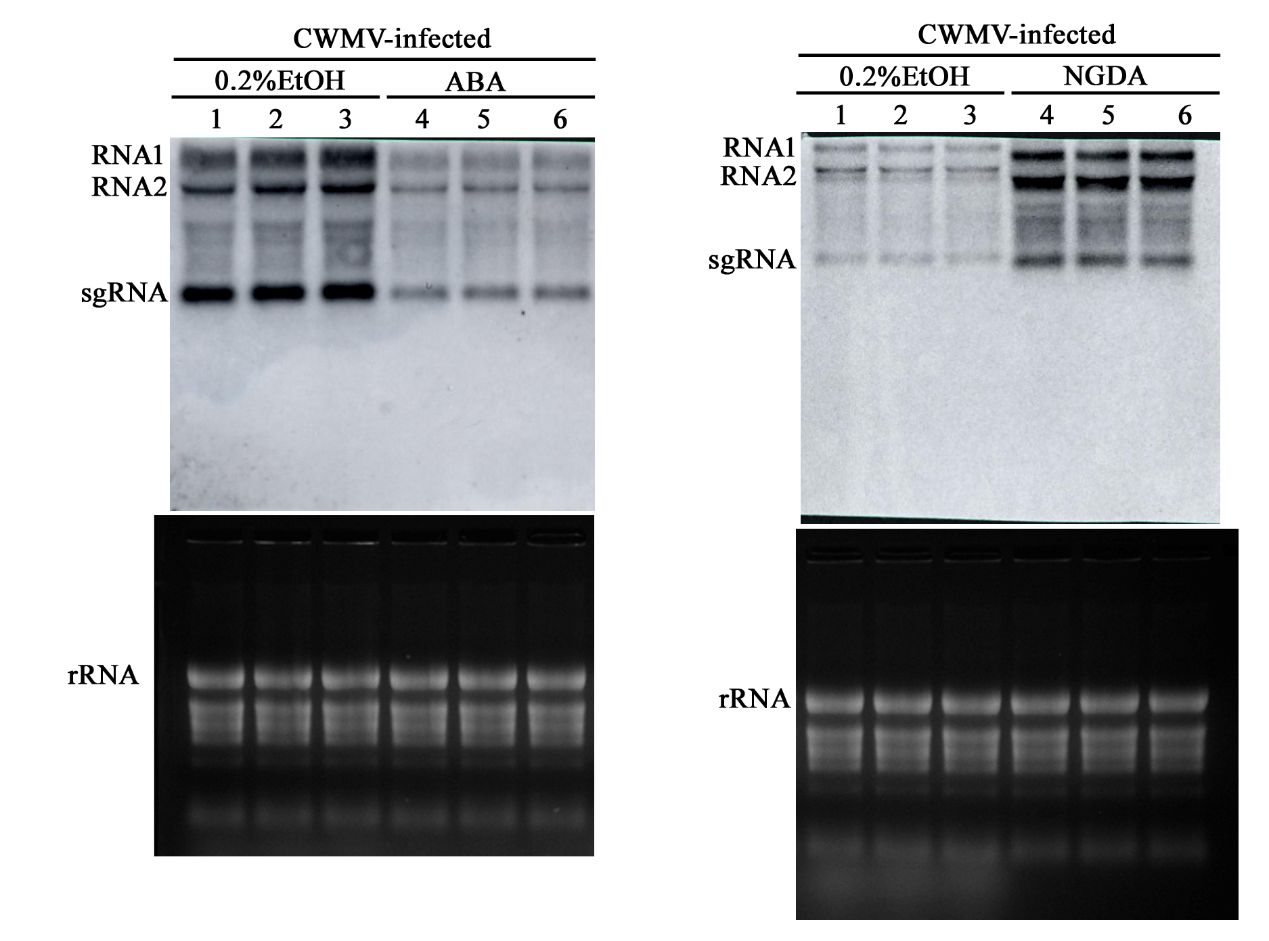


**Figure 9b** Northern blot analyses of CWMV genomic RNAs accumulations in the ABA pre-treated CWMV-inoculated plants. Ethidium bromide-stained rRNA was used as a loading control.

**Figure 9c**


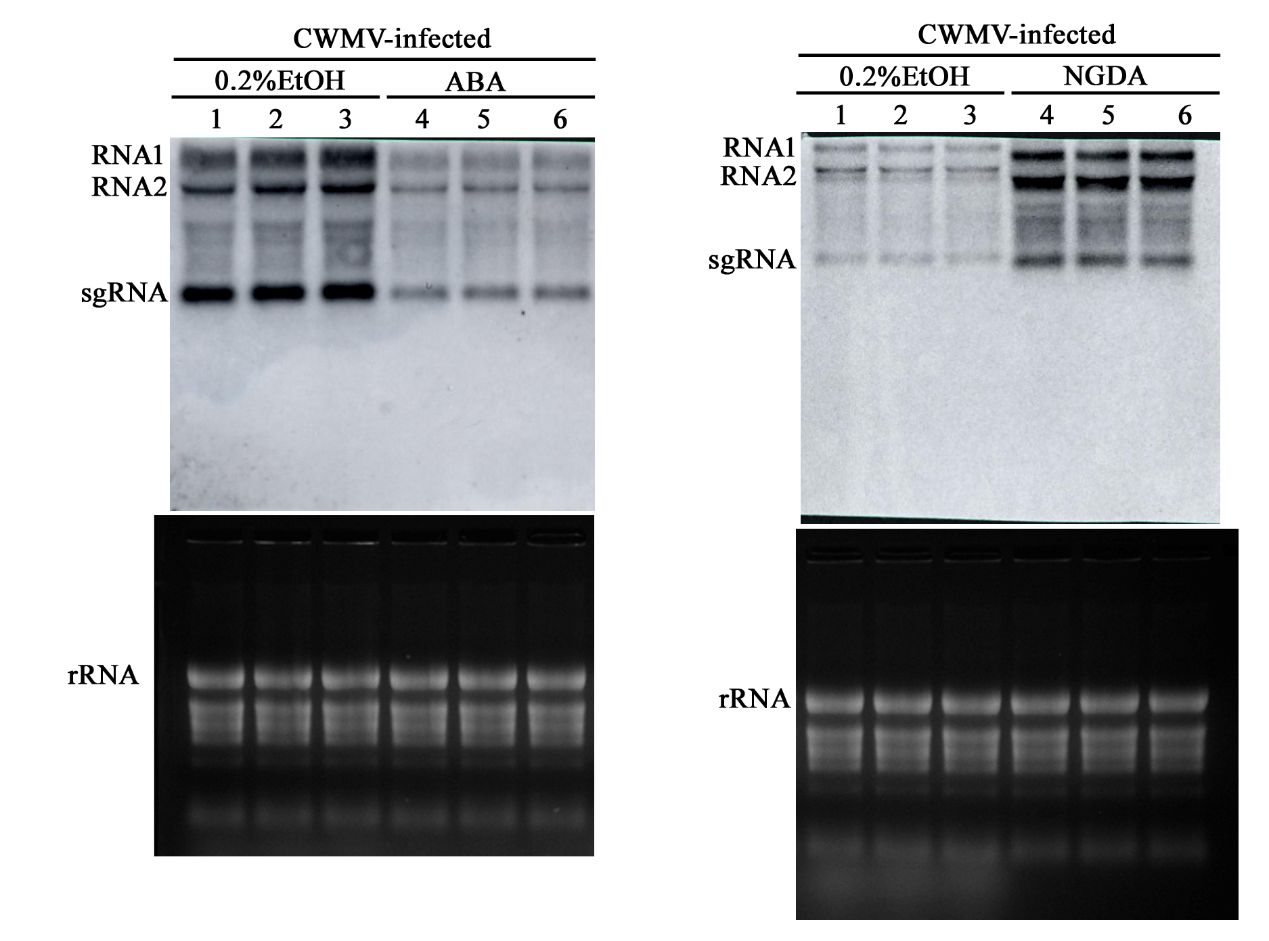


**Figure 9c** Northern blot analyses of CWMV genomic RNAs accumulations in the NDGA pre-treated CWMV-inoculated plants. Ethidium bromide-stained rRNA was used as a loading control.

**Figure 9d**


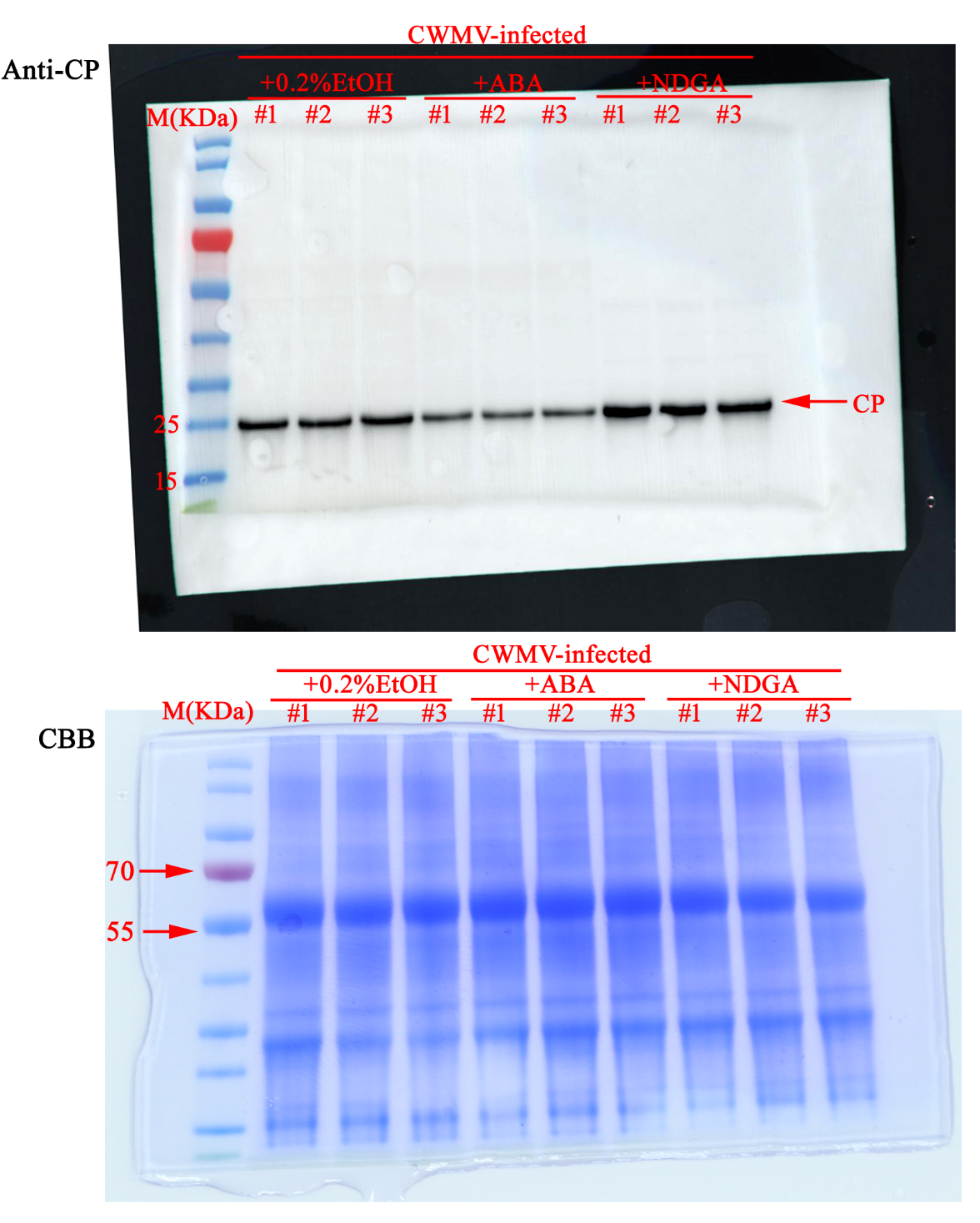


**Figure 9d** Western blot analyses of CWMV CP protein accumulation in the ABA or NDGA pre-treated CMWV-inoculated plants. CBB-stained loadings are shown at the bottom of the figure.
